# Two-speed genome evolution drives pathogenicity in fungal pathogens of animals

**DOI:** 10.1073/pnas.2212633120

**Published:** 2023-01-03

**Authors:** Theresa Wacker, Nicolas Helmstetter, Duncan Wilson, Matthew C. Fisher, David J. Studholme, Rhys A. Farrer

**Affiliations:** ^a^Medical Research Council Centre for Medical Mycology at the University of Exeter, Exeter EX4 4QD, United Kingdom; ^b^Medical Research Council (MRC) Centre for Global Infectious Disease Analysis, Imperial College London, London W12 0BZ, United Kingdom; ^c^Department of Biosciences, University of Exeter, Exeter EX4 4QD, United Kingdom

**Keywords:** Chytridiomycosis, infectious disease, two-speed genome evolution, pathogenicity

## Abstract

The origins and evolution of virulence in amphibian-infecting chytrids *Batrachochytrium dendrobatidis* (*Bd*) and *Batrachochytrium salamandrivorans* (*Bsal)* are largely unknown. Here, we use deep nanopore sequencing of *Bsal* and comparative genomics against 21 high-quality genome assemblies that span the fungal Chytridiomycota. We discover that *Bsal* has the most repeat-rich genome of the Chytridiomycota, comprising 40.9% repetitive elements; this genome has expanded to more than 3× the length of its conspecific *Bd*, with autonomous and fully functional LTR/Gypsy elements contributing significantly to the expansion. The M36 metalloprotease virulence factors are highly expanded (*n* = 177) in *Bsal*, most of which (53%) are flanked by transposable elements, suggesting they have a repeat-associated expansion. We find enrichment upstream of M36 metalloprotease genes of three novel repeat families belonging to the repeat superfamily of LINEs that are implicated with gene copy number variations. Additionally, *Bsal* has a highly compartmentalized genome architecture, with virulence factors enriched in gene-sparse/repeat-rich compartments, while core conserved genes are enriched in gene-rich/repeat-poor compartments. Genes upregulated during infection are primarily found in the gene-sparse/repeat-rich compartment in both *Bd* and *Bsal*. Furthermore, genes with signatures of positive selection in *Bd* are enriched in repeat-rich regions, suggesting these regions are a cradle for the evolution of chytrid pathogenicity. These are the hallmarks of two-speed genome evolution, and this study provides evidence of two-speed genomes in an animal pathogen, shedding light on the evolution of fungal pathogens of vertebrates driving global declines and extinctions.

## Significance

*Batrachochytrium salamandrivorans* (*Bsal*) and its closest relative *B. dendrobatidis* (*Bd*) are fungal pathogens that threaten amphibians globally. Pathogenicity in vertebrates by species of *Batrachochytrium* is thought to have emerged from nonpathogenic and saprobic relatives over millions of years through gene expansions of secreted proteolytic enzymes families. Using deep nanopore sequencing and comparative genomics, we discover that *Batrachochytrium* genomes have undergone a repeat-driven expansion characterized by flanking repetitive elements enriched around pathogenicity genes, genes with signatures of positive selection, and genes upregulated during infection. These genomic features are the hallmarks of two-speed genomes that have to date only been described in plant pathogens. These discoveries shed new light on the evolution of fungal pathogens of vertebrates driving global declines and extinctions.

*Batrachochytrium salamandrivorans* (*Bsal*) threatens amphibians globally and is currently expanding its geographic range across Europe. For example, *Bsal* infects highly susceptible fire salamanders, with outbreaks reported in the wild in Germany, Belgium, the Netherlands, and Spain (1, 2). This ecologically important fungal pathogen belongs to the *Rhizophydiales* order of the Chytridiomycota, which includes genera with mostly saprobic free-living species as well as a small number with pathogenic niches. *Entophylctis helioformis* and *Homolaphlyctis polyrhiza* are the two closest known relatives of *Batrachochytrium*. However, unlike those amphibian pathogens, *E. helioformis* and *H. polyrhiza* are saprotrophs found on algae and leaf litter and are unable to grow on amphibian skin (3, 4).

*Bsal* likely diverged from *Batrachochytrium dendrobatidis* (*Bd*) between 30 and 115 Mya in the Late Cretaceous or early Paleogene, and both species have likely been endemic to Asian salamanders and newts (Urodela) for millions of years. Both species have expanded their ranges in recent time with *Bd* becoming globally established in the early to mid-20th Century (5), while *Bsal* emerged in the Netherlands only in 2010 and has since spread to naïve European populations (2). Since diverging, *Bd* and *Bsal* have evolved to infect different amphibian species and display different pathologies. While *Bd* is a generalist pathogen that infects all three orders of amphibian*, Bsal* has evolved as a specialist pathogen of the Urodela order (newts and salamanders) (6), yet is able to survive asymptomatically on amphibians of other orders, potentially contributing to its spread (7). While *Bd* causes hyperplasia (proliferation of cells) and hyperkeratosis (thickening of the stratum corneum), *Bsal* causes multifocal superficial erosions and deep ulcerations in the skin of its host (6). The evolutionary route to pathogenicity and the genetic mechanisms underlying host specificity and pathology in the *Batrachochytrium* genus remain largely unknown.

Evolution shapes genomes unevenly, resulting in both conserved and faster evolving genomic compartments. Varying degrees of structural and functional compartmentalization and associated genomic stability have been reported for one-speed (8–10), two-speed (11–16), and multi-speed (17) genomes, where, in the case of the latter two, signatures of evolution and selection pressures demarcate functional and structural compartments. In “two-speed genomes,” rapidly evolving genes comprise a substantial portion of the genome and are associated with an enrichment of repeat families, especially transposable elements (TEs), that are likely contributing to or driving gene variation (9, 11–16, 18, 19). In plant pathogens with two-speed genomes, these dynamic and repeat-rich compartments are enriched for genes that are upregulated in planta (12), have signatures of positive selection (20), and have undergone gene family expansions (12), while core-conserved genes are found in gene-rich and repeat-sparse compartment of relative genomic stability (13). To date, two-speed genome compartmentalization has been identified in only a few fungal and oomycete plant pathogens (12, 14, 21–24). Among the chytrids, *Synchytrium endobioticum* responsible for potato wart disease has been noted to have effector genes within repeat-rich regions (25). However, there has been no comprehensive analysis or identification of two-speed genomes among the Chytridiomycota to date, nor indeed among any fungal pathogens of animals.

Recently, we sequenced *Bsal*’s genome using paired-end Illumina reads (26), discovering it to be expanded compared to its closest relatives. We found that *Bsal* has undergone large expansions of several protein families including the M36 metalloproteases that are thought to be involved in the breakdown of amphibian skin and extracellular matrix (4). The M36 metalloproteases are also virulence factors for host invasion in dermatophytes, cleave host complement during infection with the opportunistic human fungal pathogen *Aspergillus fumigatus,* promoting immune evasion, and have been associated with pathogenicity in *Coccidioides* (27–32). The M36 metalloprotease family expansions coincide with an increase in repeat-rich regions; however, that study estimated only 17% of the *Bsal* genome assembly to be repetitive (26). We also found evidence that, unlike *Bd*, *Bsal* does not illicit a clear immune response during infection in a shared host species (26). However, the limits of exclusively short-read sequencing resulted in our previous *Bsal* genome assembly being highly fragmented and we were unable to fully explore genome evolution and resolve repeat-rich regions. Furthermore, the genomes of only four chytrid species were compared. Here, we use long-read nanopore sequencing of *Bsal* and comparative genomics against 22 species of chytrids to improve our understanding of the genome evolution of the vertebrate-infecting batrachochytrids *Bd* and *Bsal*.

## Results

### The Repeat-Driven Expansion of *Bsal*.

**Fig. 1.**
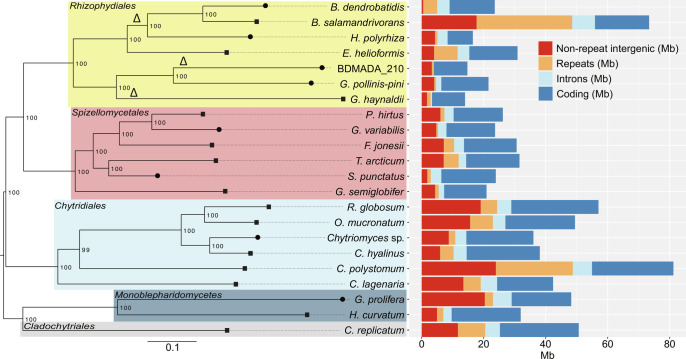
Phylogeny of 22 chytrids based on multiple alignments of 143 core orthologs (*Left*) next to genome content (*Right*). Branches with circular tips have been sequenced using short-read sequencing technologies (Illumina) and branches with square tips have been sequenced with long-read sequencing technologies (*Bsal*: Oxford Nanopore sequencing technology; all others: PacBio sequencing technology). The percentage of 1,000 ultrafast bootstrap resamplings that support the major topological elements in neighbor joining is indicated. The scale bar indicates the number of substitutions per site. Delta symbol indicates loss of RdRP.

Deep nanopore sequencing and genome assembly of *Bsal* revealed that it has undergone a substantial genome expansion compared with all known and genome-sequenced species of the *Rhizophydiales* (Fig. 1). Notably, the genome of *Bsal* is >3× longer than its closest known relative *Bd*. Our updated genome assembly (version 2; v.2) is a substantial improvement on our previous Illumina-based assembly (version 1; v.1), with a total length of 73.3 Mb across 165 supercontigs (*N_Max_* 5.6 Mb, N_50_ 0.9 Mb) compared with v.1 that is 32.6 Mb across 5,358 contigs (N_50_ 10.5 kb) (*SI Appendix*, Table S1). *Bsal*’s updated genome length elevates it to the second-largest in the Chytridiomycota fungal phyla, after *Cladochytrium polystomum* (81.2 Mb), a species that is mainly associated with aquatic plants (33–35). Our updated gene annotation also revealed slightly higher numbers of predicted protein-coding genes (*n* = 10,867 with a combined length of 16.38 Mb) and was slightly more complete (94.1% complete Benchmarking Universal Single-Copy Orthologs (BUSCO) for core conserved fungal genes) compared to the v.1 assembly (complete BUSCO = 93%). Synteny analysis indicated that there were no newly acquired chromosomes in *Bsal*’s genome compared with *Bd*, although its genome expansion relative to *Bd* was accompanied by an abundance of chromosomal rearrangements, most of which are within rather than at the ends of contigs (suggesting they are not an artefact of contig ordering) (*SI Appendix*, Fig. S1). Ploidy analysis and variant calling suggested that *Bsal* is diploid like most *Bd* isolates (36), with ~2.3 heterozygous positions per kb (*SI Appendix*, Figs. S2 and S3), which is approximately half the previously calculated rate, likely owing to the better resolution of repetitive regions and gene families. Heterozygous positions were identified throughout every chromosome in the genome, suggesting abundant allelic variation (*SI Appendix*, Fig. S4).

**Fig. 2.**
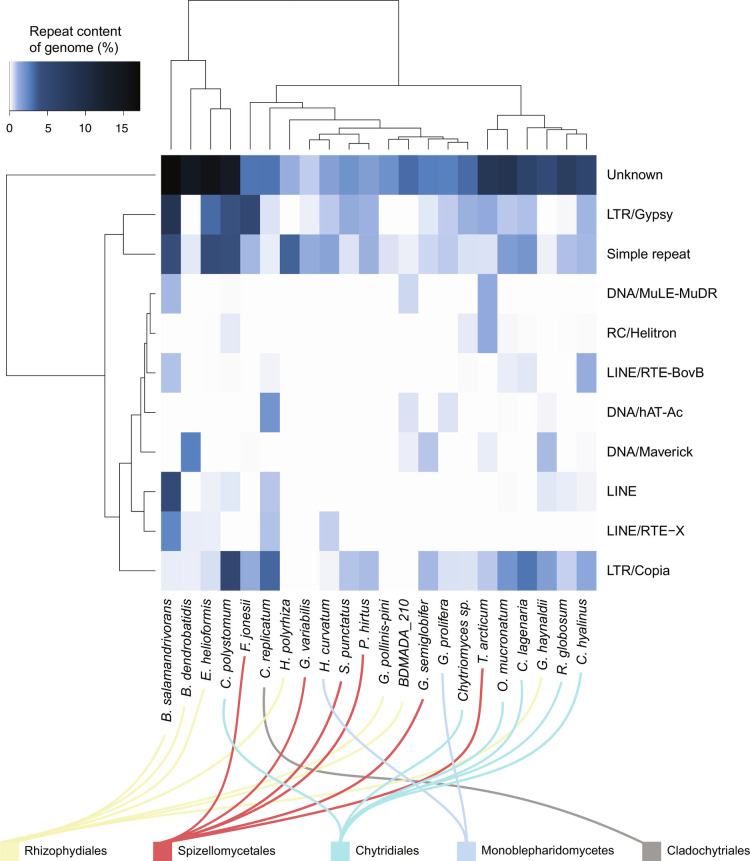
Repeat superfamily abundance in 23 Chytrids. The dendrograms are based on Euclidean distances and hierarchical clustering. Repeat families with <1% abundance in every species were excluded from the heatmap.

*Bsal* has the most repeat-rich genome of any chytrid sequenced to date, with 40.9% (30 Mb) of the genome predicted to be repetitive (*SI Appendix*, Fig. S5). *Bsal* has undergone a unique repeat-driven expansion compared with other chytrids including *Bd*, resulting in a distinct repeat family profile (Fig. 2). Repeat content across the Chytridiomycota (as a percentage of genome length) positively correlates with genome length (Spearman’s r_s_ = 0.62, *P* = 0.0019). Percent of the genome comprising TEs in the Chytridiomycota also correlates with genome length (Spearman’s r_s_ = 0.56, *P* = 0.0059). If only short-read sequenced assemblies are considered, then the correlation is not significant (r_s_ = 0.52, *P* = 0.16). However, among only long-read-based assemblies, the positive correlation remains significant (r_s_ = 0.59, *P* = 0.03). Repeat content does not correlate with assembly contiguity (N_50_) (Spearman’s r_s_ = 0.19, *P* = 0.39) or degree of fragmentation (number of contigs) (Spearman’s r_s_ = −0.022, *P* = 0.92) (*SI Appendix*, Fig. S6). Genome assembly length in the Chytridiomycota is therefore a good predictor of repeat-richness, when based on high-quality long read assemblies.

TEs, long terminal repeat (LTR), and long interspersed nuclear elements (LINE) retrotransposons are uniquely expanded in the *Bsal* genome compared with *Bd* (Fig. 2). *Bsal* has the highest overall content of TEs of any chytrid (*SI Appendix*, Fig. S7). TEs comprise 19.36% of all *Bsal* repetitive content and are not uniformly distributed in the genome but appear in clusters. Conversely, repeats in general, including simple repeats, are more uniformly distributed (*SI Appendix*, Fig. S8). *C. polystomum* (the longest genome assembly) has the second-highest proportion of TEs (12.3%). All other chytrids (excluding *Bsal* and *C. polystomum*) have <10% TEs (geometric mean: 2.96%, SD: 2.26%) indicating that TE content associates with chytrid genome expansions.

LTRs are the second most abundant repeat family in *Bsal* (6.6 Mb; 9% of genome), most of which (97%) are Gypsy elements. A large fraction of Gypsy family repeats (*n* = 636/2,958; 21.5%) have the capacity to be active (encoding the GAG structural protein, an aspartic proteinase, a reverse transcriptase (RT) and a DDE integrase). Gypsy repeats are far less common in other *Rhizophydiales* including *Bd* (4.8 kb; 0.02% of genome, none of which are fully functional or autonomous), *H. polyrhiza* (absent)*,* and *E. helioformis* (988 kb; 3.2%).

LINEs make up 6.4 Mb (8.8%) of the *Bsal* genome (Dataset S1), yet are not detected in most of the other genomes belonging to the *Rhizophydiales* including *H. polyrhiza*, BdMADA_210 (an amphibian-associated chytrid recovered from Madagascar) and *G. pollinis-pini* [a saprotrophic chytrid found in aquatic habitats (37)]. The three remaining *Rhizophydiales* species (*Bd*, *E. helioformis*, and *G. haynaldii*) have only low numbers of LINE (0.28%, 0.6%, and 0.47% of genome, respectively). We found six LINE families (*n* = 6/21; 28.6%) are capable of mobilization (encoding 1 RT and 1 apurinic endonuclease domain) (Dataset S2) (38). When considering each individual occurrence of all LINE in *Bsal’s* genome, only 5.25% (*n* = 145/2,763) have the capacity to be active (encoding ≥ 1 RT).

*Bd* has markedly fewer autonomous TEs compared with *Bsal,* no fully functional LINE or LTR families, and fewer fully functional and autonomous Class II DNA transposon families. The only LINE/RTE-X family present in *Bd* (named rnd1 family 109) encodes an RT but not the additional apurinic endonuclease required for full functionality. Together, this suggests that TEs in *Bsal* are more active in shaping its genome than in *Bd* (Dataset S2 and *SI Appendix*).

Repeat-induced point mutation (RIP) is a fungal-specific genomic mechanism for controlling TE proliferation. However, we found that *Bd* has no signatures of RIP and lacks genes for the essential proteins for RIP including the 5-C cytosine methyltransferase (RIP defective; RID), in line with previous reports (25). Similarly, we found that *Bsal* has no signatures of RIP or the RID protein in its genome (Dataset S3 and *SI Appendix*, Fig. S9). Other mechanisms to control TEs include RNAi based mechanisms including quelling (39–43). Core components of the RNAi machinery are Dicer, Argonaute/Piwi, and RNA dependent RNA polymerase (RdRP) (44). Both Dicer and Argonaute are encoded by *Bd* and *Bsal*, as well as all other chytrids investigated (Dataset S3), while RdRP homologs are not found in several chytrids including *Bd* and *Bsal*, *G. haynaldii,* BdMADA_210, and *H. polyrhiza*, all of which are members of *Rhizophydiales*. In the absence of RIP, batrachochytrids may control TE spread by post-transcriptional RNAi-based TE regulation, for instance, via double-strand RNA-mediated mRNA degradation for which RdRP is not involved (45).

### The “Two-Speed” Compartmentalized Genome of *Bsal*.

**Fig. 3.**
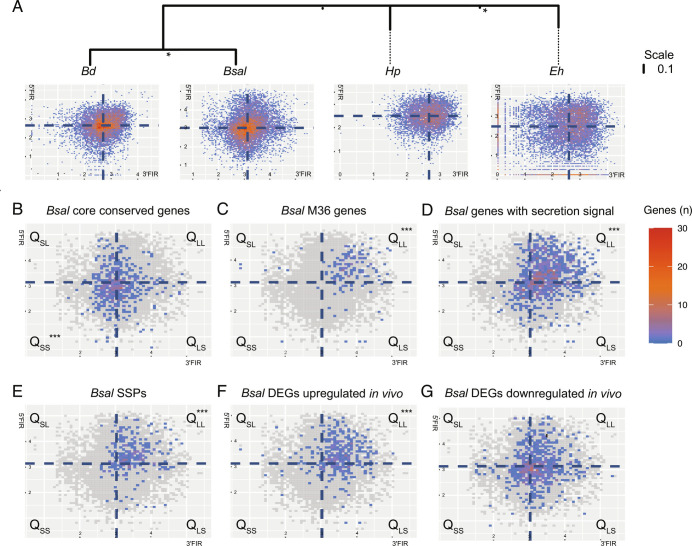
The two-speed genome of *Bsal*. (*A*) a phylogenetic tree of *Bsal* and its three closest relatives: *Bd, Hp*, and *Eh* constructed using a core ortholog multiple alignment and RAxML. Vertical branch lengths indicating the mean number of nucleotide substitutions per site. Asterisks indicate 100% bootstrap support from 1,000 replicates. Density plots of intergenic distance for all nonterminal protein coding genes are shown (measured as log_10_ length of 5’ and 3’ flanking intergenic distances; FIRs), with the median values shown in dotted blue line. (*B*–*E*) density plots of intergenic distance for *Bsal* gene categories with gene-sparse compartments designated Q_LL_ (*Upper-Right* quadrant: long 3’ and 5’ distances) and gene-rich compartments designated as Q_SS_ (*Lower-Left* quadrant: short 3’ and 5’ distances). Intermediate compartments are designated Q_SL_ and Q_LS._. Genes lacking both FIR (i.e., at contig ends) are excluded. (*B*) core-conserved genes, (*C*) M36 metalloprotease, (*D*) genes with a secretion signal, (*E*) genes encoding SSPs, (*F*) differentially expressed genes (DEGs) upregulated in vivo and (*G*) DEGs downregulated in vivo. The median intergenic distance for all genes is shown as a dotted blue line. Asterisks indicate enrichment of genes in one of the four quadrants based on the median intergenic distances (HgT, α-level = 0.01).

The analysis of chytrid intergenic distances revealed *Bsal* has a compartmentalized, bipartite genome. Disparate flanking intergenic region (FIR) lengths for several gene families and biological functions were identified across the Chytridiomycota (Fig. 3 and *SI Appendix*, Fig. S10). To assess differences between FIR lengths and gene categories, we characterized four groups or quadrants partitioned by the 5′ and 3′ median intergenic distances (short-long: Q_SL_; long-long: Q_LL_; short-short: Q_SS_ and long-short: Q_LS_) and tested for enrichment of genes using hypergeometric tests (HgTs) and χ^2^ tests. Core conserved genes (CCGs) were enriched in Q_SS_ (HgT *P* = 5.88E^−6^, χ^2^ test *P* = 7.16E^−6^). Meanwhile, several gene groups were enriched in Q_LL_, including M36 protease genes, genes encoding secreted proteins and genes encoding small secreted proteins (SSPs) according to HgT (*P* = 1.2E^−^^38^, *P* = 5.42E^−^^92^, *P* = 9.13E^−^^7^, respectively) and χ^2^ tests (*P* = 1.93E^−^^43^, *P* = 5.39E^−^^102^, *P* = 4.4E^−^^7^, respectively) (Datasets S4 and S5). The FIR length is also associated with repetitive content. Namely, *Bsal* has 3.5× more repetitive sequence content and 4.5× more TE content in 10 kb windows with one or more genes belonging to Q_LL_ compared with Q_SS_. This is consistent with gene-sparse compartments also being repeat-rich in two-speed genomes.

**Fig. 4.**
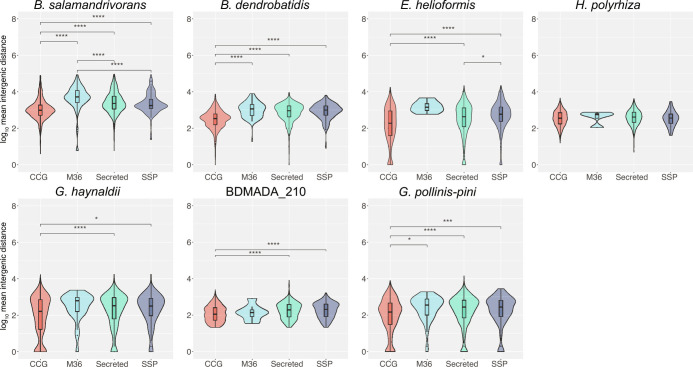
*Rhizophydiales* Intergenic Distance Analysis. Log_10_ mean intergenic distances of core-conserved genes (CCGs), SSPs (<300 amino acids and ≥4 cysteines), secreted proteins and M36 metalloproteases for seven species of *Rhizophydiales*. Boxplots indicate median and interquartile range. Statistically significant differences of log_10_ mean intergenic distances for pairs of different gene groups was determined using a Wilcoxon rank-sum test. *P*-values were Bonferroni adjusted with an α-level of 0.01 and *n* = 6. *P ≤ 0.0017, **P ≤ 0.00017, ***P ≤ 1.7E^−^^5^, ****P ≤ 1.7E^−^^6^.

FIR lengths are significantly longer for M36 proteases, genes encoding secreted proteins, and SSPs compared to overall mean intergenic distances in *Bsal* (Wilcoxon rank-sum tests: *P* = 1.42E^−^^61^, 2.45E^−^^121^ and 2.50E^−^^7^, respectively). Mean intergenic distances for M36 proteases, genes encoding secreted proteins, and SSPs are also significantly longer than CCGs (Wilcoxon rank-sum tests: *P* = 1.46E^−^^61^, 8.54E^−^^72^ and 2.4E^−^^13^, respectively). Additionally, CCGs are flanked by significantly shorter intergenic regions than the genome-wide average (Wilcoxon rank-sum test *P* = 2.03E^−^^9^) (Fig. 4). Intriguingly, most chytrids (18 species out of 23) had an enrichment of CCGs in Q_SS,_ and nearly half (10 species out of 23) had an enrichment for genes encoding proteins with secretion signals in Q_LL_ (HgT and χ^2^ test *q* < 0.01), indicating those are common features of Chytridiomycota evolution. *Bsal* has the most significant enrichment of genes with secretion signals in Q_LL_ of all the chytrids (HgT *P* = 5.42E^−^^92^, χ^2^ test *P* = 5.39E^−^^102^), while *Bd* had the second strongest enrichment (HgT *P* = 9.75E^−^^67^, χ^2^ test *P* = 4.18E^−^^74^).

Genes belonging to each of the quadrants are dispersed across the genome, and not exclusively from individual chromosomes or large sub-chromosomal or sub-telomeric regions (*SI Appendix*, Fig. S11). Similarly, M36 metalloproteases are encoded throughout the *Bsal* and *Bd* genomes (*SI Appendix*, Fig. S1). Of the 28 contigs that feature a (TTAGGG)_n_ terminal telomeric repeat, 14 feature clusters of up to 10 M36s or genes with secretion signal in those sub-telomeric regions, suggesting an association between these classes of genes and the ends of chromosomes. Six contigs overall deviate from the null hypothesis of 25% of genes populating each quadrant (χ^2^ test for goodness of fit; Dataset S6), three of which (scaffolds 94, 329, and 334) are >1 Mb in length. Twelve contigs were enriched for genes falling into one of the quadrants (HgT *P* < 0.01) including Q_LL_ (*n* = 9) and Q_SS_ (*n* = 3), and no enrichments were found for either Q_SL_ or Q_LS_. The longest stretch of consecutive Q_LL_ genes is 16 (128 kb), Q_SS_ genes is 13 (33 kb), Q_LS_ genes is 5 (25 kb), and for Q_SL_, it is only 4 (16 kb; Dataset S7).

We found several occurrences of consecutive genes with longer than median FIRs, which were enriched for genes encoding secreted proteins. The probability of *n* number of consecutive genes from a given quadrant was calculated using a discrete-time pattern Markov chain approach. In *Bsal*, this approach identified 24 genomic regions (encompassing 237 genes) with significant numbers of consecutive gene counts (*P* < 0.01) from either Q_LL_ (10 regions encompassing 99 genes) or Q_SS_ (14 regions encompassing 138 genes) (*SI Appendix*). Across all the chytrid genomes, an average of 14 significant genomic regions of consecutive genes were identified in Q_LL_ + Q_SS_, encompassing an average of 125 genes across all regions per species (Dataset S7). Of these genes found in significant consecutive regions, an average of 8.35 (10.75%) in Q_LL_ were predicted to have a secretion signal, compared with just 3.48 (5.24%) in Q_SS_. The largest discrepancy was found in the batrachochytrids: *Bsal* has 27 (*n* = 27/99; 27%) genes within significant stretches of Q_LL_ genes with secretion signals compared with just 4 (*n* = 4/99; 2.9%) in Q_SS_. Similarly, *Bd* has 65 (*n* = 65/170; 38%) genes (two of which were M36s) within significant stretches of Q_LL_ genes with secretion signals compared with just 1 (*n* = 1/88; 1.14%) in Q_SS_. Across all the chytrids, 15/23 (65% of species) had more genes with secretion signals in significant stretches of Q_LL_ genes compared with Q_SS_. These results reveal clusters of genes in the batrachochytrids with long FIRs that are enriched for encoding secretion signals (*Bsal*: HgT < 3.38E^−^^8^, *Bd*: HgT < 2.48E^−^^13^). Furthermore, this phenomenon is common, albeit less pronounced and nonsignificant, across the Chytridiomycota.

Genes with signatures of positive selection in isolates belonging to each lineage of *Bd* are enriched for long intergenic distances (*SI Appendix*, Fig. S12). Substantial sampling efforts for *Bd* have revealed five genetically diverse and largely isolated lineages (5, 46), providing an opportunity to explore fixed genetic differences between sub-populations and their associations with intergenic distances, which is an opportunity not currently available for *Bsal*. By calculating *d_N_*/*d_S_* (ω) for every *Bd* gene in an isolate representing each lineage, we discovered that genes with a signature of positive selection (ω > 1) were significantly enriched in Q_LL_ for each lineage (HgT *P* < 2.59E^−^^10^, χ^2^ test *P* < 6.5E^−^^11^) (Dataset S4). Notably, genes with ω > 1 and secretion signals were enriched in Q_LL_ for each *Bd* lineage (HgT *P* < 8.48E^−^^13^, χ^2^ test *P* < 3.64E^−^^14^). Therefore, *Bd* also has the hallmarks of a two-speed genome, despite having fewer repeats and a smaller genome than *Bsal*.

Recently, it has been shown that ricin-like B lectins play a role in the initial stages of pathogenesis in *Bsal* (30). Previous studies have also found that these lectins are expressed during exposure of *Bsal* to salamander skin (26). Of the two ricin-like B lectins identified in this study, one of them (BSLG_002240) can be found in Q_LL_, the other (BSLG_009176), is found in Q_LS_. This suggests that some virulence gene families are shared between dynamic and stable genomic regions, potentially benefiting from both evolutionary speeds.

Genes in the gene-sparse/repeat-rich compartment of the genome are enriched for those that are upregulated during infection of the Wenxien Knobby Newt (*T*. *wenxianensis*) (Q_LL_: *n* = 224; 61%; HgT *P* = 1.82E^−^^45^) compared with the gene-rich compartment (Q_SS_ = 22; 6%; HgT *P* = 1; *SI Appendix*, Figs. S13–S15 and Dataset S5) (26). This pattern is not observed for genes downregulated in vivo, where 31% of differentially expressed genes are found in Q_LL_ and 22% are found in Q_SS_ (HgT *P* = 4.34E^−^^2^ for Q_LL_, *P* = 1 for Q_SS_). In *Bsal*, 45% of genes upregulated in vivo encode secreted proteins, as opposed to 17% of the downregulated genes. This pattern was also found in *Bd* where 53% (*n* = 55) of genes upregulated during infection were found in Q_LL_ (HgT *P* = 2.35E^−^^8^) and 9% (*n* = 9) found in Q_SS_ (HgT *P* = 1). Additionally, 42% of differentially expressed *Bd* genes upregulated in vivo encode for secreted proteins, as opposed to 13% of the downregulated genes in vivo*.* Together, these results show a positive association between gene function, intergenic length, and expression levels.

**Fig. 5.**
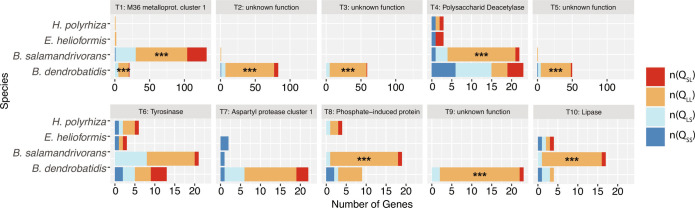
Secreted protein tribes in *Bsal*, *Bd*, *Eh*, and *Hp*. The 10 biggest secreted protein tribes identified by clustering all predicted secreted genes using MCL found in one or more of the four chytrids (*Bsal*, *Bd*, *Eh*, and *Hp*). Significance of enrichment was determined using HgTs. Q_LL_ = gene-sparse region, Q_SS_ = gene-rich region. Asterisks indicate enrichment of genes in one of the four quadrants (HgT, *P*_adjusted_ < 0.00063).

*Bsal* encodes the greatest percent of secreted proteins (9.64%; *n* = 1,047) among the Chytridiomycota. Clustering secreted proteins by amino acid sequence for *Bsal* and its three closest relatives *Bd*, *Hp*, and *Eh*) revealed 854 distinct secreted tribes, including the M36 metalloproteases tribe that is the largest (*n* = 167, of which 142 belong to *Bsal*; Tribe 1). The ten largest secreted tribes encompass nearly a quarter of all secreted proteins of *Bsal*, *Bd*, *Hp*, and *Eh* (24.08%; *n* = 593) (Dataset S8). Notable gene tribes included the M36s (Tribe 1), polysaccharide deacetylases (Tribe 4), tyrosinases (Tribe 6), aspartyl proteases (Tribe 7), phosphate-induced proteins (Tribe 8), and lipases (Tribe 10), each of which may be involved in pathogenicity. *Bsal* genes in Tribes 1, 4, 8, and 10 are enriched in Q_LL_. *Bd* genes in Tribes 1, 2, 3, 5, 7, and 9 are enriched in Q_LL_ (Fig. 5 and Dataset S4).

### M36 Metalloprotease Expansion Linked to TEs.

*Bsal* encodes the most M36 metalloproteases (*n* = 177) of any chytrid (*SI Appendix*, Fig. S1), and >5× more than *Bd* encodes (*n* = 35) (4). Of the M36 metalloproteases 77.4% are secreted, compared to only 5% of all other proteases (excluding M36 metalloproteases). Many (*n* = 63; 35%) of *Bsal*’s M36 metalloproteases are also significantly differentially expressed in vivo, most of which (*n* = 47) were upregulated in vivo. This is compared with 5/37 (14%) differentially expressed M36s encoded by *Bd* (two of which were upregulated in vitro, and three upregulated in vivo).

**Fig. 6.**
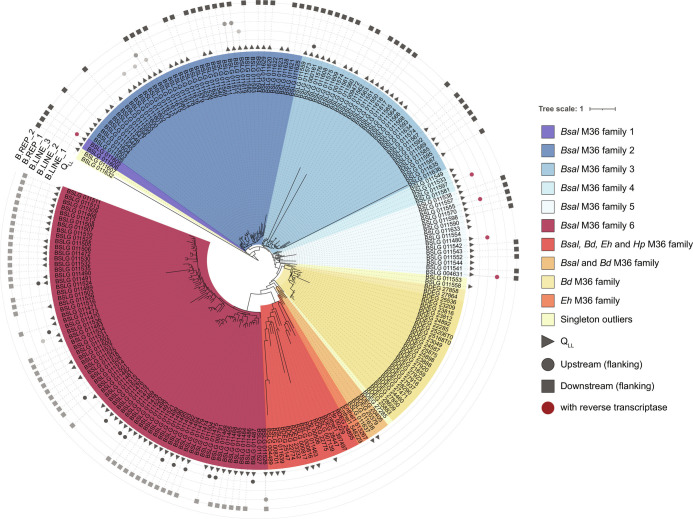
A gene tree inferred using RaxML from protein alignments of all identified M36 proteins in four chytrids (*Bsal*, *Bd*, *Eh*, and *Hp*). The branch lengths (Tree scale) indicate the mean number of nucleotide substitutions per site. Five upstream and downstream flanking repeat families are indicated as circles (B. = Batr; BatrLINE-1, 2, and 3) and squares (BatrREP-1 and 2). Genes in the gene-sparse Q_LL_ are indicated by a filled triangle shape. LINEs with a functional reverse transcriptase (RT) domain are marked in dark red. Gene IDs: Enthel1 = *E. helioformis*, *Hp* = *H. polyrhiza*, BSLG = *B. salamandrivorans* and BDEG = *B. dendrobatidis*. See *SI Appendix*, Fig. S16 for a more detailed version.

M36 metalloproteases in *Bsal* can be divided into six species-specific families (*Bsal* M36 family 1 to 6) and two more evolutionary conserved families based on sequence similarity and a gene tree (Fig. 6, *SI Appendix*, Fig. S16, and Dataset S9). Several LINE and LINE/RTE-X superfamilies are significantly enriched upstream of M36 metalloproteases. *Bsal’s* largest M36 family (family 6; *n* = 70; 77.1% secreted) was previously named *Bsal* G2M36 (26) and is flanked by 14 LINE and LINE/RTE-X families (12 BatrLINE-1, 1 BatrLINE-2 and 1 BatrLINE-3). There are only 69 total occurrences of BatrLINE-1, 2, and 3, 20% of which are flanking M36s. All other *Bsal* M36 sub-families have genes flanked by BatrLINE-1, 2, and 3 upstream (*n* = 13/107; 12.15%). Most genes  n the *Bsal* M36 family 6 are also flanked downstream by an uncharacterized repeat that we have named BatrREP-1 (*n* = 52/70; 75.7%). No other *Bsal* M36 family has a flanking BatrREP-1. Conversely, another uncharacterized repeat (BatrREP-2) flanks M36 families 1 to 5 downstream (*n* = 53/107; 49.5%). Therefore, three LINE and two uncharacterized repeat families are associated with *Bsal*’s genome expansion and specifically its M36 gene family expansion.

*Bsal* M36-associated repeats are enriched upstream and downstream of the M36 metalloprotease coding genes (Dataset S4). Specifically, BatrLINE-1, 2, and 3 are enriched upstream of M36s (*P* = 1.21E^−^^10^, 6.98E^−^^5^ and 2.41E^−^^5^, respectively) and genes encoding secreted proteins (*P* = 4.09E^−^^06^, 2.78E^−^^4^ and 7.39E^−^^6^, respectively). Similarly, BatrREP-1 and 2 are enriched downstream of M36s (*P*_BatrREP-1_ = 1.85E^−^^96^ and *P*_BatrREP-2_ = 2.20E^−^^106^) and genes encoding secreted proteins (*P*_BatrREP-1_ = 1.15E^−^^38^ and *P*_BatrREP-2_ = 1.63E^−^^49^). Most of these repeats are disproportionately found in gene-sparse/repeat-rich compartments (Q_LL_) of the genome (*P*_BatrLINE-1_ = 9.81E^−^^5^, *p*_BatrLINE-2_ = 1.69E^−^^3^, *P*_BatrREP-1_ = 5.34E^−^^9^, *P*_BatrREP-2_ = 4.08E^−^^13^). *Bd*’s genome has a repeat family (rnd1 family 109) with sequence similarity to BatrLINE-1, suggesting an ancestral origin, although it is not classified as a LINE. Furthermore, rnd1 family 109 is only present upstream of one M36 metalloprotease and eight genes encoding secreted proteins in *Bd*. No sequence similarity was identified for BatrREP-1 or 2 in *Bd*. Only 66/734 (9%) *Bsal* repeat families had sequence similarity to *Bd’s* repeat families suggesting that *Bsal*’s two-speed genome is largely driven by repeat families that have emerged since it diverged with *Bd*.

*Bsal*’s M36 associated BatrLINE-1 consensus sequence encodes an RT and apurinic endonuclease domain (Dataset S2), suggesting that it is autonomous and fully functional. BatrLINE-1 was not differentially expressed during infection (*SI Appendix* and Dataset S10) (38). A total of 271/825 (32.8%) individual occurrences of BatrLINE-1 have an RT domain, although only 39 (4.7%) are fully functional (encoding the additional apurinic endonuclease). Only a single BatrLINE-1 with a RT is found upstream of a M36 metalloprotease (M36 family 1). Most BatrLINE-1 upstream of M36 metalloproteases shares sequence similarity and diverge from one internal node of their gene tree. However, one BatrLINE-1 with an RT falls outside that group (*SI Appendix*, Fig. S17). The consensus sequences of BatrLINE-2 and BatrLINE-3 feature a RT domain, but no apurinic endonuclease. Of their individual occurrences in the genome, 13.6% and 0.6%, respectively, possess a recognizable RT domain. Thus, most of the M36-associated repeats are no longer active.

## Discussion

The chytridiomycosis panzootic has been identified as one of the key drivers of global amphibian declines, contributing to earth’s sixth mass extinction (47). Since the discovery of *Bd* (48) and more recently *Bsal* (6), efforts have been made to understand their evolution and mechanisms of pathogenicity and virulence. Here, we show clear evidence that *Bsal*’s genome has been shaped by adaptation to the amphibian skin environment, resulting in large and diverse families of proteolytic enzymes for skin and extracellular matrix destruction (26, 49–53). We assemble and annotate an improved *Bsal* assembly and perform comparative genomics across the Chytridiomycota, discovering that both *Bsal* and *Bd* have the hallmarks of two-speed genomes, shedding light on their adaptation to the amphibian host.

*Bsal* has an extremely repeat-rich genome (40.9%) compared with most fungal species, which typically range from ~5 to 35% (54). Many of *Bsal’*s repeats are TEs (19.36%) which underpin the genome expansions described in other species including the fungal wheat pathogen *Zymoseptoria tritici,* the barley powdery mildew, *Blumeria graminis* f. sp. *hordei,* the oomycete causative agent of potato blight, *Phytophthora infestans*, and the symbiotic fungus *Cenococcum geophilum* (55–58). TEs are abundant in the genomes of several fungal and oomycete pathogens. For example, TEs make up 36% of the genome for plant pathogen *Leptosphaeria maculans*, 64% of the genome for *B. graminis* and 74% for *P. infestans* (55, 56, 59). TEs in *Bsal*’s closest known relative *Bd*, however, only comprises 3.37% of its genome, suggesting TEs have expanded recently in *Bsal* and that the differences in genome size in the batrachochytrids is at least partly caused by TE expansion in *Bsal*.

The most abundant TE family in *Bsal* is the Class 1 LTR/Gypsy, which is almost absent in *Bd* (0.02%). 98.5% of all LTR/Gypsy repeat occurrences belong to repeat families whose consensus sequences feature all necessary domains rendering them autonomous and fully functional. More than 1/5 (21.5%) individual occurrences of LTR/Gypsy have the capacity to be active and therefore retain the ability to continue to contribute to further genome expansion. In contrast, humans have half a million Class 1 TE’s, of which <0.1% are functional/mobile due to various mutations (60). *Bsal*’s closest known relative *Bd*’s has a significantly smaller genome that does not contain any LTR families that feature domains necessary to mobilize, suggesting TE’s have become activated in *Bsal* since their divergence, and contributed to *Bsal's* genome expansion. Indeed, LTR/Gypsy elements have been previously identified as a driver of genome expansion and adaptation along environmental gradients and under stress conditions (61–65). A positive correlation of TE content (but not overall repeat content) with genome size has been previously described in the Dikarya fungal subkingdom (66). In this study, we found a strong correlation between both TE and repeat content with genome size in the Chytridiomycota fungal phyla.

Several filamentous fungal plant pathogens and oomycetes have bipartite genome architectures with gene-sparse/repeat-rich compartments enriched with effector genes (such as those coding for secreted proteins that function outside of the organism they were synthesized in), acting as cradles of adaptive evolution (9, 11–13, 67). These repeat-rich/gene-sparse compartments are associated with higher evolvability and genome plasticity and are often enriched in TEs, feature structural and copy number variations and are enriched with genes under positive selection (11, 12, 14, 23, 59, 68–70). Conversely, gene-rich/repeat-sparse compartments are enriched in CCGs (12). This two-speed genome therefore could potentially provide an efficient evolutionary solution for high evolvability in some parts and conservation in others, providing genome plasticity while reducing the risk of excessive deleterious mutations in essential genes.

We identify and define this bipartite “two-speed” genome compartmentalization based on an approach that acknowledges that dynamic compartments are both a structural and functional categorization. We show that a bipartite genome architecture can be defined based on both the structural compartmentalization of groups of genes into gene rich/repeat sparse and repeat rich/gene sparse, as well as a functional compartmentalization reflected by enrichment of genes with respective functional categories: conserved genes and putative effector genes with signatures of positive selection, respectively. We propose a set of statistical methods to identifying such a compartmentalization, including enrichment tests, tests to determine statistically significant differences in median intergenic distances, as well as sliding-window and discrete-time pattern Markov chain calculations. Our methods were specifically designed to identify dichotomous genomes organizations; however, it is feasible to adapt them for multi-speed genomes using alternative thresholds and permutations.

Here we show that the batrachochytrids also have a bipartite “two-speed” genome compartmentalization, which is especially pronounced in *Bsal*. The gene-sparse compartments in both batrachochytrids are enriched in putative effector genes encoding secreted proteins, M36 metalloproteases, most of which are secreted and which have been associated with host invasion and pathogenicity in, *e.g.*, dermatophytes, and ricin B-like lectins, shown to be implicated with chemotaxis, adhesion, and the early stages of a pathogenesis (26, 27, 29–31, 62, 71–73). In *Bd*, gene-sparse compartments are enriched for genes with signatures of positive selection, indicating that the gene-sparse compartment is a hot spot of adaptive evolutionary processes in batrachochytrids, potentially contributing to the high mutation rates observed in *Bsal* with substitution rates of up to 8.25 × 10^−5^ per site per year (74). That *Bd* has not undergone the same repeat-driven genome expansion as *Bsal*, suggests that genome compartmentalization can result from alternative, potentially epigenetic mechanisms such as chromatin organization. Indeed, such processes may also underpin *Bsal*’s genome architecture.

We discovered further evidence of genomic compartmentalization in genes upregulated during infection (of the Wenxien Knobby Newt) that are enriched in the dynamic, gene-sparse compartment in both *Bsal* and *Bd*, with *Bsal* showing a markedly stronger enrichment. This evolutionary signature was not identified for genes downregulated in vivo. Furthermore, greater numbers of genes encoding secreted proteins were upregulated in vivo than those downregulated in vivo. The relevance of secretion for virulence in host–pathogen interactions is widely recognized and further highlights the relevance of dynamic compartments in virulence and pathogenicity (75).

M36 metalloproteases are thought to break down the amphibian skin and extracellular matrix during infection of the host (4). However, this function needs to still be experimentally confirmed in amphibians. Another function of M36 metalloproteases is immune evasion by cleaving complement of humans in *A. fumigatus* and they have been shown to be important for host invasion in dermatophytes, providing strong evidence for their role as virulence factors (28, 29, 31, 76). Here, we describe the largest expansion of *Bsal* M36 metalloproteases in any species, and discover large gene families of *Bsal* M36 metalloproteases (such as family 6) are enriched for flanking LINE.

LINEs are recognized as a source of gene duplications and implicated in genetic novelty where duplicated genes can evolve new functions (38, 77). A unique feature of *Bd* and *Bsal* compared with the other chytrids was the enrichment of three LINE families and two additional repeat families around any class of genes (in this case, and excitingly M36 metalloproteases that are putative virulence factors), which is a further hallmark of two-speed genomes. Interestingly, LINE found upstream of M36 metalloproteases in *Bsal* seem to have lost their functional domains, while other insertions of this LINE family retain functionality and autonomy. Possible hypotheses to explain the lack of functional domains include: 1) the loss preceded duplication or 2) natural selection independently inactivated each LINE instance, after duplication. In either case, LINE repeats could provide regions of homology for mitotic ectopic recombination, a known source of gene duplications (78). If the loss of functionality followed duplication, this might reflect strong selective pressure for these particular LINE insertions to be silenced, as it has been shown that genes flanked by TEs are often transcriptionally repressed and upregulation of some of the M36 metalloproteases flanked by this LINE family is observed during infection (66).

In fungi, repeats in genomes are targeted for mutation via the repeat induced point mutation (RIP) mechanism, which protects the genome from duplications and TE proliferation (79). In *Bsal* and *Bd*, no recognizable RIP machinery or signatures can be found*.* The absence of RIP is generally associated with a uniform distribution of TEs and an erosion of their compartmentalization. This uniform distribution of TEs has been found in genomes lacking a compartmentalized structure before (so-called one-speed genomes) (9, 80). The observed compartmentalization of TEs in *Bsal*, especially in the context of the uniform distribution of repeats overall, might be achieved by histone modification and methylation or by post-transcriptional, “quelling”-like silencing, as homologs of RNAi components are present both in *Bsal* and *Bd* (79, 81). In humans, autophagy is another post-transcriptional mechanism with which TE transcripts are degraded (82). Post-transcriptional silencing, however, does not readily explain the observed degradation of RT domains in LINE found upstream of M36 metalloproteases, suggesting that there might be other TE silencing mechanisms present in *Bsal*. The association of genes coding for secreted M36 metalloproteases with TEs and their uneven distribution in *Bsal* suggests that TEs are actively and passively shaping its genome architecture, as well as driving higher evolvability of compartments enriched in virulence factors (13, 14, 59, 67, 68, 70, 80, 83).

The stark difference between *Bsal*’s TE expansion (including active elements) relative to *Bd*, and indeed all other chytrid investigated, remains enigmatic, especially given no difference in RIP and RNAi machinery in the batrachochytrids. Possible explanations include additional undescribed repeat silencing mechanisms that differ between *Bd* and *Bsal* or different selection pressures (*e.g.,* bottlenecks, gene drift, positive selection acting on TE-associated species-specific gene family expansions).

For the first time, we discover that two important pathogens of vertebrates, *Bsal* and *Bd*, have the hallmarks of two-speed genomes, which may underpin their transition to pathogenicity from presumed saprobic ancestors. Furthermore, we establish that the bipartite genome architectures found in several plant pathogens are a more general outcome of evolution and not limited to plant pathogens. In the batrachochytrids, these two-speed genome processes have clearly contributed to genome architecture, with genes likely to be involved in pathogenicity enriched within genomic compartments that allow for rapid adaptive evolution.

## Materials and Methods

Full details are given in *SI Appendix*. Briefly, *Bsal* zoosporangia and zoospores were cultured in tryptone-gelatin hydrolysate-lactose (TGhL) broth in cell culture flasks at 18 °C. High-molecular weight DNA for Nanopore sequencing was obtained by a customized cetyltrimethylammonium bromide (CTAB) extraction procedure (84). Two independent sequencing libraries were constructed, one with long unfragmented DNA, one with DNA fragmented to 12 kb with a gTube (520079, Covaris). The two libraries were loaded onto a single PromethION (FLO-PRO002, type R9.4.1) flowcell and sequenced. Nanopore reads were trimmed using PoreChop v.0.2.3_seqan2.1 (85) with default parameters, and filtered where <500 bp or average read quality >10 using NanoFilt v.2.6.0 (86). Canu v.1.8 (87) was used to assemble reads ≥ 100 kb (~13× coverage) with stopOnLowCoverage = 0.5, genomeSize = 0.6 g and minReadLength = 500. Gene annotation on the repeat masked V2 assembly was guided by our previous 14.4 Gb *Bsal* in vitro RNAseq (NCBI BioProject PRJNA326249) using the Braker2 (88) pipeline (parameters–fungus, –softmasking).

Chytrid genomes were compared to additional chytrid genomes downloaded from the MycoCosm portal of the US Department of Energy (DOE) Joint Genome Institute (JGI) (89). Single copy orthologs were identified between chytrids using the Synima (90) pipeline with Orthofinder, and aligned using MUSCLE v3.8.31 (91). A maximum likelihood tree was constructed using IQ-Tree v1.6.12 (92) with the LG amino acid substitution model (the best fitting model according to ProtTest v3.4.2 (93) with 1000 ultrafast bootstraps, and visualized using Figtree v1.4.4 with midpoint rooting. Repeat content was identified using Repeatmodeller v.2.0.1(94) with rmblast v.2.10.0+ and Tandem Repeat Finder v.4.09 (95), RepeatScout v.1.06 (96) and RepeatMasker v.4.0.5 (97). To identify autonomous and nonautonomous TEs, repeats were scanned for functional domains against the Database of Protein Families (PFAM; release 35.0) and Conserved Domains Database (CDD; release 3.19) databases, using hmmsearch and rpsblast, respectively (98, 99, 100 and 101). Flanking intergenic distance was calculated for all nonterminal protein coding genes. For each chytrid species, the median distance was used to define four quadrants. For *Bd*, Genome Analysis Toolkit (GATK) v.4.1.2.0 (102) was used to call variants and *dN*/*dS* values for each gene in each lineage determined using the yn00 program of PAML (103). To analyze gene expression and the expression of consensus repeat families, we obtained our *Bsal* in vitro RNAseq reads (NCBI BioProject PRJNA326249), *Bd* in vitro RNAseq reads (PRJNA326253), and *Bd* or *Bsal* infected *Tylototriton wenxianensis* (PRJNA300849). Replicates for *Bd* and *Bsal* were compared separately using the Trinity v2.13.2 differential expression pipeline (104).

## Supplementary Material

Appendix 01 (PDF)

Dataset S01 (XLSX)

Dataset S02 (XLSX)

Dataset S03 (XLSX)

Dataset S04 (XLSX)

Dataset S05 (XLSX)

Dataset S06 (XLSX)

Dataset S07 (XLSX)

Dataset S08 (XLSX)

Dataset S09 (XLSX)

Dataset S10 (XLSX)

Dataset S11 (TXT)

Dataset S12 (TXT)

Dataset S13 (TXT)

Dataset S14 (TXT)

Dataset S15 (TXT)

Dataset S16 (TXT)

## Data Availability

Raw *B. salamandrivorans* sequences are deposited at GenBank under Bioproject PRJNA666901 (https://www.ncbi.nlm.nih.gov/bioproject/PRJNA666901). The new genome assembly and annotations for *Bsal* are deposited at GenBank under Bioproject PRJNA311566 (https://www.ncbi.nlm.nih.gov/bioproject/PRJNA311566; assembly name Batr_sala_V2). Repeat sequences are saved in Datasets S11–S16. Scripts used for windows analysis and enrichment analysis are uploaded to https://github.com/rhysf/2speed_genomes and https://bitbucket.org/Theresa_42/wackeretal_2022_bsal_2speedgenome.

## References

[1] W. Beukema , Landscape epidemiology of *Batrachochytrium salamandrivorans*: Reconciling data limitations and conservation urgency. Ecol. Appl. **31**, e2342 (2021). 10.1002/eap.2342 33817953

[2] A. Martel , Wildlife disease. Recent introduction of a chytrid fungus endangers Western Palearctic salamanders. Science **346**, 630–631 (2014). 25359973 10.1126/science.1258268 PMC5769814

[3] J. E. Longcore, P. M. Letcher, T. Y. James, *Homolaphlyctis polyrhiza* gen. et sp. nov., a species in the *Rhizophydiales* (Chytridiomycetes) with multiple rhizoidal axes. Mycotaxon **118**, 433–440 (2012).

[4] S. Joneson, J. E. Stajich, S.-H. Shiu, E. B. Rosenblum, Genomic transition to pathogenicity in chytrid fungi. PLoS Pathog. **7**, e1002338 (2011). 22072962 10.1371/journal.ppat.1002338 PMC3207900

[5] S. J. O’Hanlon , Recent Asian origin of chytrid fungi causing global amphibian declines. Science **360**, 621–627 (2018). 29748278 10.1126/science.aar1965 PMC6311102

[6] A. Martel , *Batrachochytrium salamandrivorans sp. nov.* causes lethal chytridiomycosis in amphibians. Proc. Natl. Acad. Sci. U.S.A. **110**, 15325–15329 (2013). 24003137 10.1073/pnas.1307356110 PMC3780879

[7] S. More , Risk of survival, establishment and spread of *Batrachochytrium salamandrivorans* (Bsal) in the EU. EFSA J. **16**, e05259 (2018). 32625888 10.2903/j.efsa.2018.5259 PMC7009437

[8] B. Schwessinger , A near-complete haplotype-phased genome of the dikaryotic wheat stripe rust fungus *Puccinia striiformis* f. sp. tritici Reveals High Interhaplotype Diversity. mBio **9**, e02275-17 (2018). 29463659 10.1128/mBio.02275-17 PMC5821087

[9] L. Frantzeskakis, S. Kusch, R. Panstruga, The need for speed: Compartmentalized genome evolution in filamentous phytopathogens. Mol. Plant Pathol. **20**, 3–7 (2019). 30557450 10.1111/mpp.12738 PMC6430476

[10] R. Gourlie , The pangenome of the wheat pathogen *Pyrenophora tritici-repentis* reveals novel transposons associated with necrotrophic effectors ToxA and ToxB. BMC Biol. **20**, 239 (2022). 36280878 10.1186/s12915-022-01433-w PMC9594970

[11] B. J. Haas , Genome sequence and analysis of the Irish potato famine pathogen *Phytophthora infestans*. Nature **461**, 393–398 (2009). 19741609 10.1038/nature08358

[12] S. Raffaele , Genome evolution following host jumps in the irish potato famine pathogen lineage. Science **330**, 1540–1543 (2010). 21148391 10.1126/science.1193070

[13] S. Dong, S. Raffaele, S. Kamoun, The two-speed genomes of filamentous pathogens: Waltz with plants. Curr. Opin. Genet. Dev. **35**, 57–65 (2015). 26451981 10.1016/j.gde.2015.09.001

[14] L. Faino , Transposons passively and actively contribute to evolution of the two-speed genome of a fungal pathogen. Genome Res. **26**, 1091–1100 (2016). 27325116 10.1101/gr.204974.116 PMC4971763

[15] B. M. Tyler , *Phytophthora* genome sequences uncover evolutionary origins and mechanisms of pathogenesis. Science **313**, 1261–1266 (2006). 16946064 10.1126/science.1128796

[16] S. Raffaele, S. Kamoun, Genome evolution in filamentous plant pathogens: Why bigger can be better. Nat. Rev. Microbiol. **10**, 417–430 (2012). 22565130 10.1038/nrmicro2790

[17] L. Fokkens , The multi-speed genome of *Fusarium oxysporum* reveals association of histone modifications with sequence divergence and footprints of past horizontal chromosome transfer events. bioRxiv. 10.1101/465070 (Accessed 29 September 2022).

[18] K. H. Lamour , Genome sequencing and mapping reveal loss of heterozygosity as a mechanism for rapid adaptation in the vegetable pathogen *Phytophthora capsici*. Mol. Plant Microbe Interact. **25**, 1350–1360 (2012). 22712506 10.1094/MPMI-02-12-0028-R PMC3551261

[19] M. Gijzen, Runaway repeats force expansion of the *Phytophthora infestans* genome. Genome Biol. **10**, 241 (2009). 19843349 10.1186/gb-2009-10-10-241 PMC2784315

[20] A. Sánchez-Vallet , The genome biology of effector gene evolution in filamentous plant pathogens. Annu. Rev. Phytopathol. **56**, 21–40 (2018). 29768136 10.1146/annurev-phyto-080516-035303

[21] D. E. Torres, U. Oggenfuss, D. Croll, M. F. Seidl, Genome evolution in fungal plant pathogens: Looking beyond the two-speed genome model. Fungal Biol. Rev. **34**, 136–143 (2020).

[22] D. J. Winter , Repeat elements organise 3D genome structure and mediate transcription in the filamentous fungus *Epichloë festucae*. PLoS Genet. **14**, e1007467 (2018). 30356280 10.1371/journal.pgen.1007467 PMC6218096

[23] C. Plissonneau, A. Stürchler, D. Croll, The evolution of orphan regions in genomes of a fungal pathogen of wheat. mBio **7**, e01231-16 (2016), 10.1128/mbio/7/5/e01231-16. 27795389 PMC5082898

[24] Q. Wang , Characterization of the two-speed subgenomes of *Fusarium graminearum* reveals the fast-speed subgenome specialized for adaption and infection. Front. Plant Sci. **8**, 140 (2017). 28261228 10.3389/fpls.2017.00140 PMC5306128

[25] B. van de Vossenberg , Comparative genomics of chytrid fungi reveal insights into the obligate biotrophic and pathogenic lifestyle of *Synchytrium endobioticum*. Sci. Rep. **9**, 8672 (2019). 31209237 10.1038/s41598-019-45128-9 PMC6572847

[26] R. A. Farrer , Genomic innovations linked to infection strategies across emerging pathogenic chytrid fungi. Nat. Commun. **8**, 14742 (2017). 28322291 10.1038/ncomms14742 PMC5364385

[27] O. Jousson , Multiplication of an ancestral gene encoding secreted fungalysin preceded species differentiation in the dermatophytes *Trichophyton* and *Microsporum*. Microbiology **150**, 301–310 (2004). 14766908 10.1099/mic.0.26690-0

[28] J. Li, K.-Q. Zhang, Independent expansion of zincin metalloproteinases in onygenales fungi may be associated with their pathogenicity. PLoS One **9**, e90225 (2014). 24587291 10.1371/journal.pone.0090225 PMC3938660

[29] R. Shende , *Aspergillus fumigatus* conidial metalloprotease Mep1p cleaves host complement proteins. J. Biol. Chem. **293**, 15538–15555 (2018). 30139746 10.1074/jbc.RA117.001476 PMC6177592

[30] Y. Wang , Epidermal galactose spurs chytrid virulence and predicts amphibian colonization. Nat. Commun. **12**, 5788 (2021). 34608163 10.1038/s41467-021-26127-9 PMC8490390

[31] F. Brouta , Secreted metalloprotease gene family of *Microsporum canis*. Infect. Immun. **70**, 5676–5683 (2002). 12228297 10.1128/IAI.70.10.5676-5683.2002 PMC128366

[32] X. Zhang , Metalloprotease genes of *Trichophyton mentagrophytes* are important for pathogenicity. Med. Mycol. **52**, 36–45 (2014). 23859078 10.3109/13693786.2013.811552

[33] M. J. Powell, P. M. Letcher, J. E. Longcore, W. H. Blackwell, Zopfochytrium is a new genus in the Chytridiales with distinct zoospore ultrastructure. Fungal Biol. **122**, 1041–1049 (2018). 30342620 10.1016/j.funbio.2018.08.005

[34] B. Czeczuga, B. Mazalska, A. Godlewska, Fungi and fungus-like organisms (Straminipila) on fruit tree petals floating in water. Biol. Lett. **44**, 41–50 (2007).

[35] B. Czeczuga, E. Muszyńska, A. Godlewska, B. Mazalska, Aquatic fungi and straminipilous organisms on decomposing fragments of wetland plants. Mycol. Balc. **4**, 31–44 (2007).

[36] R. A. Farrer , Chromosomal copy number variation, selection and uneven rates of recombination reveal cryptic genome diversity linked to pathogenicity. PLoS Genet. **9**, e1003703 (2013). 23966879 10.1371/journal.pgen.1003703 PMC3744429

[37] L. Pm , Ultrastructural and molecular analyses of Rhizophydiales (Chytridiomycota) isolates from North America and Argentina. Mycol. Res. **112**, 759–782 (2008). 18501579 10.1016/j.mycres.2008.01.025

[38] T. Wicker , A unified classification system for eukaryotic transposable elements. Nat. Rev. Genet. **8**, 973–982 (2007). 17984973 10.1038/nrg2165

[39] N. Romano, G. Macino, Quelling: Transient inactivation of gene expression in *Neurospora crassa* by transformation with homologous sequences. Mol. Microbiol. **6**, 3343–3353 (1992). 1484489 10.1111/j.1365-2958.1992.tb02202.x

[40] V. Fulci, G. Macino, Quelling: Post-transcriptional gene silencing guided by small RNAs in *Neurospora crassa*. Curr. Opin. Microbiol. **10**, 199–203 (2007). 17395524 10.1016/j.mib.2007.03.016

[41] B. J. Loftus , The genome of the basidiomycetous yeast and human pathogen *Cryptococcus neoformans*. Science **307**, 1321–1324 (2005). 15653466 10.1126/science.1103773 PMC3520129

[42] S. Torres-Martínez, R. M. Ruiz-Vázquez, The RNAi Universe in fungi: A varied landscape of small RNAs and biological functions. Annu. Rev. Microbiol. **71**, 371–391 (2017). 28657888 10.1146/annurev-micro-090816-093352

[43] C. Lax , The evolutionary significance of RNAi in the fungal kingdom. Int. J. Mol. Sci. **21**, 9348 (2020). 33302447 10.3390/ijms21249348 PMC7763443

[44] F. E. Nicolás, R. M. Ruiz-Vázquez, Functional diversity of RNAi-associated sRNAs in fungi. Int. J. Mol. Sci. **14**, 15348–15360 (2013). 23887655 10.3390/ijms140815348 PMC3759863

[45] P. Stein, P. Svoboda, M. Anger, R. M. Schultz, RNAi: Mammalian oocytes do it without RNA-dependent RNA polymerase. RNA **9**, 187–192 (2003). 12554861 10.1261/rna.2860603 PMC1370384

[46] R. A. Farrer , Multiple emergences of genetically diverse amphibian-infecting chytrids include a globalized hypervirulent recombinant lineage. Proc. Natl. Acad. Sci. U.S.A. **108**, 18732–18736 (2011). 22065772 10.1073/pnas.1111915108 PMC3219125

[47] B. C. Scheele , Amphibian fungal panzootic causes catastrophic and ongoing loss of biodiversity. Science **363**, 1459–1463 (2019). 30923224 10.1126/science.aav0379

[48] L. Berger , Chytridiomycosis causes amphibian mortality associated with population declines in the rain forests of Australia and Central America. Proc. Natl. Acad. Sci. U.S.A. **95**, 9031–9036 (1998). 9671799 10.1073/pnas.95.15.9031 PMC21197

[49] A. Papkou , The genomic basis of Red Queen dynamics during rapid reciprocal host–pathogen coevolution. Proc. Natl. Acad. Sci. U.S.A. **116**, 923–928 (2019). 30598446 10.1073/pnas.1810402116 PMC6338873

[50] M. C. Fisher, F. Pasmans, A. Martel, Virulence and pathogenicity of chytrid fungi causing amphibian extinctions. Annu. Rev. Microbiol. **75**, 673–693 (2021). 34351790 10.1146/annurev-micro-052621-124212

[51] A. Tellier, S. Moreno-Gámez, W. Stephan, Speed of adaptation and genomic footprints of host-parasite coevolution under arms race and trench warfare dynamics. Evol. Int. J. Org. Evol. **68**, 2211–2224 (2014). 10.1111/evo.12427 24749791

[52] D. E. Cook, C. H. Mesarich, B. P. H. J. Thomma, Understanding plant immunity as a surveillance system to detect invasion. Annu. Rev. Phytopathol. **53**, 541–563 (2015). 26047564 10.1146/annurev-phyto-080614-120114

[53] M. A. Brockhurst , Running with the Red Queen: The role of biotic conflicts in evolution. Proc. R. Soc. B Biol. Sci. **281**, 20141382 (2014). 10.1098/rspb.2014.1382 PMC4240979 25355473

[54] J. Wöstemeyer, A. Kreibich, Repetitive DNA elements in fungi (Mycota): Impact on genomic architecture and evolution. Curr. Genet. **41**, 189–198 (2002). 12172959 10.1007/s00294-002-0306-y

[55] S. Raffaele, S. Kamoun, Genome evolution in filamentous plant pathogens: Why bigger can be better. Nat. Rev. Microbiol. **10**, 417–430 (2012). 22565130 10.1038/nrmicro2790

[56] P. D. Spanu , Genome expansion and gene loss in powdery mildew fungi reveal tradeoffs in extreme parasitism. Science **330**, 1543–1546 (2010). 21148392 10.1126/science.1194573

[57] U. Oggenfuss , A population-level invasion by transposable elements triggers genome expansion in a fungal pathogen. Elife **10**, e69249 (2021). 34528512 10.7554/eLife.69249 PMC8445621

[58] M. Peter , Ectomycorrhizal ecology is imprinted in the genome of the dominant symbiotic fungus *Cenococcum geophilum*. Nat. Commun. **7**, 12662 (2016). 27601008 10.1038/ncomms12662 PMC5023957

[59] J. Grandaubert , Transposable element-assisted evolution and adaptation to host plant within the *Leptosphaeria maculans-Leptosphaeria biglobosa* species complex of fungal pathogens. BMC Genom. **15**, 891 (2014). 10.1186/1471-2164-15-891 PMC4210507 25306241

[60] G. Bourque , Ten things you should know about transposable elements. Genome Biol. **19**, 199 (2018). 30454069 10.1186/s13059-018-1577-z PMC6240941

[61] Y. Wang, W. Liang, T. Tang, Constant conflict between Gypsy LTR retrotransposons and CHH methylation within a stress-adapted mangrove genome. New Phytol. **220**, 922–935 (2018). 29762876 10.1111/nph.15209

[62] S.-J. Zhang, L. Liu, R. Yang, X. Wang, Genome size evolution mediated by Gypsy retrotransposons in Brassicaceae. Genomics Proteomics Bioinf. **18**, 321–332 (2020). 10.1016/j.gpb.2018.07.009 PMC7801240 33137519

[63] G. Wos, R. R. Choudhury, F. Kolář, C. Parisod, Transcriptional activity of transposable elements along an elevational gradient in *Arabidopsis arenosa*. Mob. DNA **12**, 7 (2021). 33639991 10.1186/s13100-021-00236-0 PMC7916287

[64] B. Pietzenuk , Recurrent evolution of heat-responsiveness in Brassicaceae COPIA elements. Genome Biol. **17**, 209 (2016). 27729060 10.1186/s13059-016-1072-3 PMC5059998

[65] H. S. Marcon , Transcriptionally active LTR retrotransposons in Eucalyptus genus are differentially expressed and insertionally polymorphic. BMC Plant Biol. **15**, 198 (2015). 26268941 10.1186/s12870-015-0550-1 PMC4535378

[66] R. Castanera , Transposable elements versus the fungal genome: Impact on whole-genome architecture and transcriptional profiles. PLoS Genet. **12**, e1006108 (2016). 27294409 10.1371/journal.pgen.1006108 PMC4905642

[67] S. Raffaele, S. Kamoun, Genome evolution in filamentous plant pathogens: Why bigger can be better. Nat. Rev. Microbiol. **10**, 417–430 (2012). 22565130 10.1038/nrmicro2790

[68] L. Schrader, J. Schmitz, The impact of transposable elements in adaptive evolution. Mol. Ecol. **28**, 1537–1549 (2019). 30003608 10.1111/mec.14794

[69] D. Croll, B. A. McDonald, The accessory genome as a cradle for adaptive evolution in pathogens. PLoS Pathog. **8**, 3 (2012). 10.1371/journal.ppat.1002608 PMC3343108 22570606

[70] T. Rouxel , Effector diversification within compartments of the *Leptosphaeria maculans* genome affected by repeat-induced point mutations. Nat. Commun. **2**, 202 (2011). 21326234 10.1038/ncomms1189 PMC3105345

[71] F. Gao , Deacetylation of chitin oligomers increases virulence in soil-borne fungal pathogens. Nat. Plants **5**, 1167–1176 (2019). 31636399 10.1038/s41477-019-0527-4

[72] Q. Xu , A polysaccharide deacetylase from *Puccinia striiformis f. sp. tritici* is an important pathogenicity gene that suppresses plant immunity. Plant Biotechnol. J. **18**, 1830–1842 (2020). 31981296 10.1111/pbi.13345 PMC7336287

[73] K. Ramakrishna Rao, E. R. B. Shanmugasundaram, Biochemical and genetical studies on host parasite relationship: Role of tyrosinase in pathocenicity and host resistance of two mutants of *fusarium vasinfectum* Atk. Mycopathol. Mycol. Appl. **42**, 299–304 (1970). 4993002 10.1007/BF02051959

[74] M. Kelly , Diversity, multifaceted evolution, and facultative saprotrophism in the European *Batrachochytrium salamandrivorans* epidemic. Nat. Commun. **12**, 6688 (2021). 34795258 10.1038/s41467-021-27005-0 PMC8602665

[75] M. J. Bradshaw , Delivering the goods: Fungal secretion modulates virulence during host–pathogen interactions. Fungal Biol. Rev. **36**, 76–86 (2021).

[76] X. Zhang , Metalloprotease genes of Trichophyton mentagrophytes are important for pathogenicity. Med. Mycol. **52**, 36–45 (2014). 23859078 10.3109/13693786.2013.811552

[77] S. Chen, B. H. Krinsky, M. Long, New genes as drivers of phenotypic evolution. Nat. Rev. Genet. **14**, 645–660 (2013). 23949544 10.1038/nrg3521 PMC4236023

[78] A. Gusa, S. Jinks-Robertson, Mitotic recombination and adaptive genomic changes in human pathogenic fungi. Genes **10**, 901 (2019). 31703352 10.3390/genes10110901 PMC6895784

[79] E. Gladyshev, Repeat-induced point mutation and other genome defense mechanisms in fungi. Microbiol. Spectr. **5**, 5.4.02 (2017). 10.1128/microbiolspec.funk-0042-2017 PMC5607778 28721856

[80] L. Frantzeskakis , Signatures of host specialization and a recent transposable element burst in the dynamic one-speed genome of the fungal barley powdery mildew pathogen. BMC Genom. **19**, 381 (2018). 10.1186/s12864-018-4750-6 PMC5964911 29788921

[81] Ö. Deniz, J. M. Frost, M. R. Branco, Regulation of transposable elements by DNA modifications. Nat. Rev. Genet. **20**, 417–431 (2019). 30867571 10.1038/s41576-019-0106-6

[82] H. Guo , Autophagy supports genomic stability by degrading retrotransposon RNA. Nat. Commun. **5**, 5276 (2014). 25366815 10.1038/ncomms6276

[83] J. Bao , PacBio sequencing reveals transposable elements as a key contributor to genomic plasticity and virulence variation in *Magnaporthe oryzae*. Mol. Plant **10**, 1465–1468 (2017). 28838703 10.1016/j.molp.2017.08.008

[84] B. Schwessinger, J. P. Rathjen, “Extraction of high molecular weight DNA from fungal rust spores for long read sequencing” in Wheat Rust Diseases: Methods and Protocols, S. Periyannan, Ed. (Springer, New York, 2017), pp. 49–57. 10.1007/978-1-4939-7249-4_5 28856640

[85] R. R. Wick, L. M. Judd, C. L. Gorrie, K. E. Holt, Completing bacterial genome assemblies with multiplex MinION sequencing. Microb. Genom. **3**, e000132 (2017). 29177090 10.1099/mgen.0.000132 PMC5695209

[86] W. De Coster, S. D’Hert, D. T. Schultz, M. Cruts, C. Van Broeckhoven, NanoPack: Visualizing and processing long-read sequencing data. Bioinformatics **34**, 2666–2669 (2018). 29547981 10.1093/bioinformatics/bty149 PMC6061794

[87] S. Koren , Canu: Scalable and accurate long-read assembly via adaptive k-mer weighting and repeat separation. Genome Res. **27**, 722–736 (2017). 28298431 10.1101/gr.215087.116 PMC5411767

[88] K. J. Hoff, A. Lomsadze, M. Borodovsky, M. Stanke “Whole-genome annotation with BRAKER” in Methods in Molecular Biology, M. Kollmar, Ed. (Humana, New York, NY, ed. 1962, 2019), pp. 62–95. 10.1007/978-1-4939-9173-0_5 PMC6635606 31020555

[89] I. V. Grigoriev , MycoCosm portal: Gearing up for 1000 fungal genomes. Nucleic Acids Res. **42**, D699–D704 (2014). 24297253 10.1093/nar/gkt1183 PMC3965089

[90] R. A. Farrer, Synima: A synteny imaging tool for annotated genome assemblies. BMC Bioinformatics **18**, 507 (2017). 29162056 10.1186/s12859-017-1939-7 PMC5697234

[91] R. C. Edgar, MUSCLE: A multiple sequence alignment method with reduced time and space complexity. BMC Bioinformatics **5**, 113 (2004). 15318951 10.1186/1471-2105-5-113 PMC517706

[92] L.-T. Nguyen, H. A. Schmidt, A. von Haeseler, B. Q. Minh, IQ-TREE: A fast and effective stochastic algorithm for estimating maximum-likelihood phylogenies. Mol. Biol. Evol. **32**, 268–274 (2015). 25371430 10.1093/molbev/msu300 PMC4271533

[93] D. Darriba, G. L. Taboada, R. Doallo, D. Posada, ProtTest 3: Fast selection of best-fit models of protein evolution. Bioinformatics **27**, 1164–1165 (2011). 21335321 10.1093/bioinformatics/btr088 PMC5215816

[94] J. M. Flynn , RepeatModeler2 for automated genomic discovery of transposable element families. Proc. Natl. Acad. Sci. U.S.A. **117**, 9451–9457 (2020). 32300014 10.1073/pnas.1921046117 PMC7196820

[95] G. Benson, Tandem repeats finder: A program to analyze DNA sequences. Nucleic Acids Res. **27**, 573–580 (1999). 9862982 10.1093/nar/27.2.573 PMC148217

[96] A. L. Price, N. C. Jones, P. A. Pevzner, De novo identification of repeat families in large genomes. Bioinformatics **21**, 351–358 (2005). 10.1093/bioinformatics/bti1018 15961478

[97] A. Smith, R. Hubley, P. Green, RepeatMasker Open-4.0 (2015).

[98] Z. Yang, PAML 4: Phylogenetic analysis by maximum likelihood. Mol. Biol. Evol. **24**, 1586–1591 (2007). 17483113 10.1093/molbev/msm088

[99] B. J. Haas , *De novo* transcript sequence reconstruction from RNA-Seq: Reference generation and analysis with Trinity. Nat. Protoc. **8**, 1494–1512 (2013). 23845962 10.1038/nprot.2013.084 PMC3875132

[100] C. Camacho , BLAST+: Architecture and applications. BMC Bioinform. **10**, 421 (2009). 10.1186/1471-2105-10-421 PMC2803857 20003500

[101] J. Mistry , Pfam: The protein families database in 2021. Nucleic Acids Res. **49**, D412–D419 (2021). 33125078 10.1093/nar/gkaa913 PMC7779014

[102] S. Lu , CDD/SPARCLE: The conserved domain database in 2020. Nucleic Acids Res. **48**, D265–D268 (2020). 31777944 10.1093/nar/gkz991 PMC6943070

[103] S. R. Eddy, Accelerated profile HMM searches. PLoS Comput. Biol. **7**, e1002195 (2011). 22039361 10.1371/journal.pcbi.1002195 PMC3197634

[104] A. McKenna , The genome analysis toolkit: A MapReduce framework for analyzing next-generation DNA sequencing data. Genome Res. **20**, 1297–1303 (2010). 20644199 10.1101/gr.107524.110 PMC2928508

