# Supplementary material for: Two-speed genome evolution drives pathogenicity in fungal pathogens of animals
Source: Proc Natl Acad Sci U S A. 2023 Jan 3;120(2):e2212633120. doi: 10.1073/pnas.2212633120 (PMC9926174; doi:10.1073/pnas.2212633120)
Supplement: Supplementary file 1 — Appendix 01 (PDF) [file pnas.2212633120.sapp.pdf]

PNAS
www.pnas.org

Supplementary Information for

Two-speed genome evolution drives pathogenicity in fungal pathogens of animals

Theresa Wacker[1], Nicolas Helmstetter[1], Duncan Wilson[1], Matthew C. Fisher[2], David J. Studholme[3], Rhys A. Farrer[1,*]

[1]Medical Research Council Centre for Medical Mycology at the University of Exeter, Exeter, United Kingdom

[2]MRC Centre for Global Infectious Disease Analysis, Imperial College London, London, United Kingdom

[3]Biosciences, University of Exeter, Exeter, United Kingdom

*Corresponding author: Rhys A. Farrer

Email: r.farrer@exeter.ac.uk

This PDF file includes:

Results

Genome assembly QC and correlations with repeat content (page 2)
Variant calling and quadrant analysis (page 3)
Autonomous and fully functional Class I and II transposable elements in *Bsal* (page 3)
RIP indices indicate absence of RIP (page 5)
Differential expression analysis (page 5)

Material & Methods

Sequencing and library preparation (page 7)
Genome assembly and quality control (page 8)
Gene annotation (page 9)
Chytridiomycota genomic and phylogenetic analysis (page 10)
Repeat Analysis (page 11)
Analysis of RIP activity and RNAi machinery (page 13)
Genome compartmentalization analysis (page 14)
Differential expression analysis (page 16)
Figures S1 to S18 (page 18)
Table S1 to S5 (page 32)
Legends for Datasets S1 to S16 (page 35)
SI References (page 41)

Other supplementary materials for this manuscript:

Datasets S1 to S16

Supplementary Results

Genome assembly QC and correlations with repeat content

Bsal assembly V2 (Canu default with Pilon polishing) does not have evidence for large internal duplications based on duplication analysis (**Figure S18; SI Genome assembly and quality control; Table S1**) suggesting it is an accurate haploid assembly. Smudgeplot (for kmer sizes 21 and 30, cutoffs chosen by the Smudgeplot cutoff function; **Figure S2**) and allele frequencies (**Figure S3**) suggest that *Bsal* has an entirely or predominantly diploid genome.

N_{50} and number of contigs are useful metrics for assembly quality (1), and were therefore used to compare the chytrid genome assemblies. However, these metrics, along with different sequencing technologies (long *vs.* short read sequencing) may be predictors or bias the characterization of repeat content. To investigate this, we compared each of these metrics and outputs for correlation (**Figure S6**). We found that N_{50} did not correlate with predicted repeat content for chytrid genome assemblies sequenced with short read (Spearman's $r_s = -0.2$, $p = 0.61$) or long read sequencing technology (Spearman's $r_s = 0.024$, $p = 0.94$). Number of contigs representing assembly fragmentation was also not correlated with repeat content for chytrid genome assemblies sequenced with short read (Spearman's $r_s = 0.14$, $p = 0.75$) or long read sequencing technologies (Spearman's $r_s = 0.43$, $p = 0.12$).

We found that repeat content across the Chytridiomycota has a positive monotonic correlation with genome length when not controlling for sequencing technique. When the sequencing technology (long *vs.* short read sequencing) is taken into consideration, a moderate, but insignificant, correlation is found for short read sequenced genomes (Spearman's $r_s = 0.52$, $p = 0.16$). For long read sequenced genomes, however, a positive monotonic correlation between genome size and repeat content is found (Spearman's $r_s = 0.59$, $p = 0.03$), suggesting that when repeats are accurately resolved, genome length in the Chytridiomycota is a predictor of repeat-richness.

Variant calling and quadrant analysis

We identified 167,709 heterozygous positions (2.3 per kb) across the *Bsal* genome, which is about half the previously predicted amount of 4.96 per kb (2), perhaps owing to the better resolution and separation of repetitive regions and gene families. These positions fell across every chromosome in the genome, supporting *Bsal* being diploid (**Figure S4**).

Using the custom discrete-time pattern Markov chain approach (https://github.com/rhysf/2speed_genomes), we identified significant stretches of genes belonging to Q_{LL} or Q_{SS} for each of the chytrid genomes. The two most significant of these in *Bsal* were scaffold 334 with 15 consecutive genes in Q_{LL} (total genes on contig = 380, p = 2.19E^{-6}) and scaffold 320 with 16 consecutive genes in Q_{LL} (total genes on contig = 122, p = 2.77E^{-6}). The 15 consecutive Q_{LL} genes on scaffold 334 included only 1 gene encoding a secreted protein (Tribe 536, with a kelch4 galactose oxidase central domain). The 16 consecutive Q_{LL} genes on scaffold 320 included 6 genes encoding secreted proteins (three belonged to Tribe 31 (unknown function), and two belonged to Tribe 17 with a S1-P1 nuclease domain).

Autonomous and fully functional Class I and II transposable elements in *Bsal*

For a transposable element (TE) to remain actively transposing, it must encode several genes (3). To explore the activity of TEs, we searched for a range of PFAM and Conserved Domains Database (CDD) domains known to be present in active TEs (**Dataset S2**), as well as the reverse transcriptase (RT) gene. Of the 21 LINE and 42 LTR families found in *Bsal*, we found 28.6% of LINEs (n = 6, including BatrLINE-1) and 35.7% of LTRs (n = 15) have the potential to be fully functional (e-value < 1E^{-3} for both Pfam hmmsearch and rspblast CDD search). LINEs were considered to be capable of mobilization if they feature at least one RT and one apurinic endonuclease domain (3). LTRs were considered to be capable of mobilization if they feature domains for GAG, a structural protein for virus-like particles, and for POL, which encodes an aspartic proteinase (AP), RT, and DDE integrase (INT) (3). We found that 90.5% of *Bsal*'s LINE repeat families and 97.6% of *Bsal*'s LTR families have a recognizable RT domain (**Dataset S2**). Notably, all LTR repeat families considered to be fully functional and autonomous (based on their consensus) belong to Gypsy elements. Of the 20 Class II

DNA transposon families found in *Bsal*, 14 (70%) have a recognizable transposase domain. Of these, 6 belong to the MuLE-MuDR class, 4 to hAT-Tag1 and 4 to TcMar-Pogo.

DNA transposons were considered autonomous and functional based on the presence of a transposase only.

Of the LINE/ LINE/RTE-X families enriched around M36 metalloproteases, BatrLINE-1 (RepeatModeler name = rnd-2 family 2), LINE family rnd-1 family 18 and LINE/RTE-X family rnd-5 family 125 found upstream of *Bsal*'s M36 metalloproteases are autonomous and fully functional. While the number of BatrLINE-1 upstream of M36 metalloproteases is significantly enriched (p = 1.21E^{-10}), rnd-5 family 125 and rnd-1 family 18 narrowly failed to reach the Bonferroni corrected significance in hypergeometric tests (HgTs;p = 1.76E^{-3} and 6.17E^{-4}, respectively) but did reach significance in the χ^2-test (p = 7.52E^{-5} and 1.88E^{-5}, respectively; **Dataset S4**). Other autonomous, fully functional LINE families flanking the M36 metalloproteases are rnd-1 family 186 (downstream) and rnd-4 family 7(upstream; neither had significant enrichment). Additionally, the autonomous (with RT) LINE/RTE-X families BatrLINE-2 (rnd-3 family 12) and BatrLINE-3 (rnd-4 family 12) are significantly enriched upstream of M36 metalloproteases based on HgTs (p = 6.98E^{-5} and 2.41E^{-5}, respectively) and χ^2-tests (p = 9.65E^{-8} and 6.25E^{-13}, respectively). Other autonomous repeat families flanking M36s with RT or transposase are LTR/Gypsy rnd1 family 39, LINE families rnd-1 family 29 and rnd-1 family18, LINE/RTE-X family rnd-1 family 27, LINE/RTE-BovB rnd-1 family 186 and DNA/MuLE-MuDR family rnd-1 family 422 (all with no significant enrichment).

Bd has markedly less autonomous and no fully functional LINE or LTR families compared with *Bsal* (**Dataset S2**). Only 28.6% (n = 4/14) *Bd*'s Class II DNA transposon families are fully functional/autonomous and have a transposase. There is only one LINE/RTE-X family present in *Bd* (rnd-1 family 109), which is the family homologous to B. LINE-1 in *Bsal*, and this family remains autonomous with a RT in *Bd*, however, with no additional apurinic endonuclease, which would render it fully functional. None of the 6 LTR repeat families in *Bd* have any recognizable RT domains. Two of the LTR/Copia repeat families feature GAG and integrase, and in one case RNaseH, but all have lost the RT and AP domain, which would allow them to be fully functional. It appears that

less TEs in *Bsal* have been inactivated and degraded than in *Bd*, with several families still being fully capable of active transposition. Only Class II DNA transposons, employing a cut-and-paste mechanism and not a copy-and-paste mechanism, have still families remaining that are capable of active transposition in *Bd.*

RIP indices indicate absence of RIP

Softwares RIPcal and RIPper applied to the genome assembly of *Bsal* suggested that RIP signatures are absent (4–7). The average RIP affected substrate index was 1.33 in *Bsal* using RIPper's sliding window approach (< 0.7 considered RIP affected). Similarly, the RIP affected product index equaled 0.77 in *Bsal* based on the sliding window approach (< 0.8 indicates a region that is not RIP affected, while > 1.1 represents a RIP signature). The composite index value of *Bsal*'s genome was -0.56 (< 0 indicates the absence of RIP). These values are very close to those calculated based on dinucleotide frequencies determined using the alignment-based approach of RIPcal: the substrate index of the average dinucleotide frequencies in repeat family alignments was 1.28 and the product index was 0.84. Focusing on LINE repeats only, the substrate index value was 1.37 and the product index value 0.59. For LTR repeat families only, they were 1.23 and 0.67, respectively.

Differential expression analysis

To determine if our differential expression analysis suffered from cross-kingdom false positive alignments, we aligned the datasets that included amphibian *Tw* (*Tw* uninfected, *Bd* infecting *Tw* and *Bsal* infected *Tw*) to another amphibian that has a genome assembly (the North American Bullfrog *Lithobates catesbeianus; Lc).* These datasets had ~6% nucleotides from sequencing data aligning to *Lc*, while chytrid only datasets (*Bd in vitro* and *Bsal in vitro*) had < 0.44% nucleotides from sequencing data aligning to *Lc*. Of those chytrid reads that aligned to *Lc*, only 17 of the 22,238 annotated *Lc* genes had any coverage (**Table S3**). Five *Lc* genes had chytrid RNA align from both *Bd* and *Bsal*, which all had < 1/3rd of the gene covered. The remaining 12 *Lc* genes were covered by either *Bd* reads of *Bsal* reads, and all had a very low breadth and depth of coverage. Those 17 *Lc* genes with any chytrid RNA aligning to them were BLASTp (or BLASTn for AB205_0169950) to the NCBI nr database, revealing they are highly

conserved genes such as tubulin, actin, homeobox genes, HSP70 and elongation factors (**Table S4**). Therefore, the accuracy of chytrid differential expression was largely unaffected by the amphibian 'contaminant' from the *ex vivo* datasets.

The *Bsal* repeat family (consensus sequence) for BatrLINE-1 is down-regulated *in vivo* (10 days post infection of *T. wenxienses* (2)) compared with *in vitro* (logFC -2.53; FDR 0.0002; **Dataset S10**). However, of the 825 individual occurrences of BatrLINE-1 LINE repeats (formerly rnd2 family2), only 13 are differentially expressed, of which 12 are downregulated *in vivo* (**Dataset S10)**. BatrLINE-2 (LINE/RTE-X; formerly rnd3 family12) is not differentially expressed, neither are the unknown repeat families BatrREP-1 and BatrREP-2 (formerly rnd1 family182 and rnd1 family405). Greater numbers of repeat families are downregulated *in vivo* (n = 75) than upregulated (n = 48). Only 76.2% and 58.6% of repeat families (excluding low complexity and simple repeats) had reads aligned to them in the *in vitro* and *in vivo* datasets, respectively (**Table S5)**. This might be because the RNAseq samples were prepared using Poly-A selection that enriches for protein encoding mRNA. Of the repeat families that are enriched around secreted proteins (**Dataset S4**), only BatrLINE-3 (LINE/RTE-X; formerly rnd-4 family 12) and the unknown repeat families rnd1 family 69, rnd1 family 221 and rnd-1 family 122 are downregulated and upregulated *in vivo*, respectively (BatrLINE-3 down *in vivo*: logFC -2.41, FDR 5.52E^{-4}; rnd-1 family 69 down *in vivo*: logFC -3.2, FDR 9E^{-6}; rnd1 family 221 down *in vivo*: logFC 6.6, FDR 8.08E^{-4}; rnd-1 family 122 up *in vivo*: logFC 4.5, FDR 6.65E^{-7}; **Dataset S10**). While rnd-1 family122 and rnd-1 family 221 have no detectable conserved protein domains, rnd-1 family 69 has a AKnT-like protein domain, which belongs to the cytochrome P450 superfamily (cd11036). This domain has not been previously recognized as a domain of TEs.

Of the 10 most upregulated *Bsal* repeat families *in vivo*, only rnd-1 family 271 features a PFAM domain which is of unknown function (PF17023, DUF5098). In the 10 most downregulated repeat families *in vivo*, only rnd-1 family 159 has assigned PFAM domains (PF13391 and PF18020), which are a HNH endonuclease domain and a TIG domain in plexin, respectively. HNH endonucleases can be found fused to a reverse transcriptase in group II introns, which a target-primed retrotransposons (8, 9). Without further recognizable domains associated with a RT, it is unlikely that this repeat family can autonomously transpose.

Material and Methods

Sequencing and library preparation

Bsal zoosporangia and zoospores were cultured in tryptone-gelatin hydrolysate-lactose (TGhL) broth in cell culture flasks at 18°C. 200ml of 6 days old cultures were harvested and centrifugated at 1700g for 10 mins at 4°C. Cell pellet was washed with ice cold water and snap frozen in liquid nitrogen. High–molecular weight DNA for Nanopore sequencing was obtained by a customized cetyltrimethylammonium bromide (CTAB) extraction procedure (10, 11) with the modification of using RNase A (T3018, NEB) instead of RNase T1. Briefly, cell pellet was ground with a mortar and pestle in liquid nitrogen with 2g of sand, followed by lysis with CTAB, two-step purification with phenol/chloroform/isoamyl alcohol and precipitation with isopropanol. Care was taken to avoid DNA shearing (cut off tips, no heating of samples). DNA concentration was checked using the Qubit BR assay (Invitrogen) and DNA size range profile was checked by Tapestation gDNA screentape (Agilent).

Two independent sequencing libraries were constructed, one with long unfragmented DNA, one with DNA fragmented to 12kb with a gTube (520079, Covaris). DNA ends were FFPE repaired and end-prepped/dA-tailed using the NEBNext FFPE DNA Repair kit (M6630, NEB) and the NEBNext Ultra II End-Repair/dA-tailing Module (E7546, NEB) followed by AMPure XP bead clean-up (A63882, Beckman Coulter). Adapters were ligated using the Genomic DNA by Ligation kit (SQK-LSK109, Oxford Nanopore Technologies) and NEBNext Quick T4 DNA Ligase (E6056, NEB) followed by AMPure XP bead clean-up. The two libraries were successively loaded onto a single PromethION (FLO-PRO002, type R9.4.1) flowcell. The unfragmented library was loaded first. Guppy Basecalling Software v. 3.2.8+bd67289 was used for base calling. A total of 24,402,905 reads were base called and of these 18,678,675 (76.5%) passed the quality check. Passed reads contained 63.78 Gb of DNA sequence (85% of the total DNA nucleotide bases sequenced) amounting to ~868X depth of coverage. The mean length of nanopore read was 3,415 nt, with an N_{50} of 9,248 and a Mean Read Quality of 10.2. The longest read was 318,012 nt long.

Genome assembly and quality control

Nanopore reads were trimmed using PoreChop v.0.2.3_seqan2.1 (12) with default parameters, and filtered where < 500 bp or average read quality > 10 using NanoFilt v.2.6.0 (13). Canu v.1.8 (14) was used to assemble reads ≥ 100 kb (~13X coverage) with stopOnLowCoverage=0.5, genomeSize=0.6g and minReadLength=500 (assembly name = V2 Canu default settings) or with additional parameters corMhapFilterThreshold=0.0000000002 corMhapOptions="--threshold 0.80 --num-hashes 512 --num-min-matches 3 --ordered-sketch-size 1000 --ordered-kmer-size 14 --min-olap-length 2000 --repeat-idf-scale 50" mhapMemory=60g mhapBlockSize=500 ovlMerDistinct=0.975 (assembly name = V2 Canu non-default settings). Raven v1.1.10 (15) was used to assembly all reads ≥50 kb (~83X coverage) with default parameters (assembly name = V2 Raven default settings). Medaka v.1.0.3 (https://github.com/nanoporetech/medaka; default parameters) and the trimmed nanopore reads were used for polishing. The polished assembly (V2 Canu default settings Medaka polished) and the unpolished assembly (V2 Canu default settings) were filtered for contigs ≤ 500bp and corrected with Illumina paired-end sequence data (2) using Pilon v1.2 (16).

We compared the previously published assembly for *Bsal* (assembly name = V1) (2) to our new assemblies using a variety of tools and metrics (**Table S1**). Assembly quality was assessed using Quast v.5.0.2 (17). We evaluated each assembly for pre-annotation gene completeness using Tblastn (-e 1e-10 -v 5 -b 5 -F F) to the 248 Core Eukaryotic Genes (CEG) (18) and BUSCO v4.1.1 (19) analysis (datasets eukaryote_odb10 and fungi_odb10). Reapr v1.0.18 (20) was used on the assemblies with Illumina paired-end sequence data (insert size: 441). Internal duplication was assessed by MUMMER v4.0.0beta2 (21) nucmer (parameters --coords --maxmatch –nosimplify) and plotted for visualization using mummerplot (parameters --layout --filter; **Figure S13**). Non-self-hits (>500bp and >99% identity) were flagged as possible duplications ("Duplication Analysis" in **Table S1**). Dnadiff was run for comparative quantifications of duplications and gaps identified by MUMMER. While all V2 *Bsal* genome assemblies were improvements in multiple metrics compared with V1, we chose the V2 Canu default assembly polished with Pilon only for all subsequent analysis based on high accuracy, contiguity, completeness and coverage.

Ploidy was assessed using Smudgeplot (22) (using kmc to generate kmer databases (23)) and allele frequency plots. Using the kmer histograms generated using kmc for the respective kmer size and the proposed ploidy of 2, genome length was estimated using GenomeScope (22). In case of kmer size 21, GenomeScope estimated the genome length to be 167,775,294 bp, with the length estimate not reaching significance (p = 0.7717) and the model fit reaching 31.7-58.5%. For kmer size 30, the model did not converge. Likely the highly repetitive nature of *Bsal*'s genome accounts for estimate that is more than twice the size of our assembly. We called variants using the Illumina *Bsal* reads from (2) aligned to the new genome using BWA v.0.7.4 and Pilon v1.9 (16) (parameters –diploid, --vcf) with the diploid setting, and filtered all sites labelled as 'LowCov', 'Amb' or 'Del'.

Gene annotation

Gene annotation on the repeat masked V2 assembly was guided by our previous 14.4Gb *Bsal in vitro* RNAseq (NCBI BioProject PRJNA326249) using the Braker2 (24) pipeline (parameters --fungus, --softmasking), which uses Samtools v.0.1.19-44428cd (25), Bamtools v.2.4.0 (26), Diamond v.2.0.4 (27), Genemark-ET v4.15 (28), and Augustus v.3.2.1 (29). The pipeline identified 11,929 genes, from which 92.74% core eukaryotic genes could be identified via BLASTP (e-value < $1e^{-10}$). Next, the genome was BLASTx against Swiss-Prot (30) and KEGG (31), and HMMER hmmscan (32) against PFAM (33). We ran tRNAscan (34) and RNAmmer (35) to identify non-protein-coding genes. M36 genes from the V1 assembly were blasted to the Braker2 softmasked predictions, and included in our gene set.

Gene predictions were checked for a variety of issues, including overlapping of noncoding genes, overlapping of coding genes, and the presence of in-frame stops. Genes were named according to evidence from BLASTx and HMMER in the following order of precedence: (i) Swiss-Pro (30), (ii) TIGRfam (36), and (iii) KEGG (31) (where BLASTx hits must meet the 70% identity and 70% overlap criteria to be considered a good hit and for the name to be applied). Otherwise, genes were classified as hypothetical proteins. Genes were functionally annotated by assigning PFAM (release 27) domains (37), and BLASTx for KEGG assignment (each where E-value $<1x10^{-10}$), as

well as ortholog mapping to genes of known function. SignalP 4.0 (38) and TMHMM (39) were used to identify secreted proteins and transmembrane proteins, respectively.

The protease composition of each chytrid was determined using top high scoring pairs from BLASTp searches (e-value < $1e^{-5}$) made to the file 'pepunit.lib', which is a non-redundant library of protein sequences of all the peptidases and peptidase inhibitors that are included in the MEROPS database (Release 12.1), and compared to the 447 thousand protein sequences in the 2014 version we used in our previous genomic analyses (2). All proteases with matches to M36 metalloproteases were aligned using MUSCLE v3.8.31 (40) and trimmed of excess gaps using trimAl 1.2rev59 (41) gappyout. We constructed the gene trees with RAxML v7.7.8(42) using the JTT amino acid transition model, which was visualized using iTOL v6 (43).

Secreted proteins were predicted in each of the 22 chytrid species using SignalP 4.0 with the 'eukaryote' organism type and otherwise default settings (38). These gene sequences had their secretion signal cleaved according to the predicted cleavage site, which were then BLASTp (all vs all) with default parameters, and clustered according to sequence similarity using MCL (http://micans.org/mcl/man/clmprotocols.html) with recommended inflation setting '-I 1.4'. The MCL program mcxdump was then used with default settings to output clusters. Secreted families were classified using PFAM domains (release 34.0) (33). Small secreted proteins (SSPs) were classified as those secreted proteins with <300 amino acids and >4 cysteines.

Chytridiomycota genomic and phylogenetic analysis

The genomes and gene annotation for *B. dendrobatidis* (*Bd*) JEL423, *S. punctatus* (*Sp*) and *H. polyrhiza* (*Hp*) (2) were downloaded from NCBI (BioProject PRJNA13653, PRJNA37881 and GenBank AFSM00000000 respectively) and FigShare (44). Nineteen further chytrid genomes were downloaded with permission from the Mycocosm portal of the US Department of Energy (DOE) Joint Genome Institute (JGI) (45) including *B. helicus* (46), *C. hyalinus* JEL632, *C. lagenaria* Arg66, *Chytriomyces sp. nov.* MP71, *E. helioformis* JEL805, *F. jonesii* JEL569, *G. haynaldii* MP57, *G. pollinis-pini* Arg68, *G. prolifera*(47), *G. semiglobifer* Barr 43, *G. variabilis* JEL559, *H. curvatum* SAG235-1, *O. mucronatum* JEL802, *P. hirtus* BR81, *R. globosum* JEL800 (48), *T.*

arcticum BR59, *C. replicatum* JEL714 (49) and *C. polystomum* WB228. We excluded *B. helicus* from further analysis as it had <75% complete BUSCOs, suggesting the assembly or gene calls are incomplete.

Single copy orthologs were identified between chytrids using the Synima (50) pipeline with Orthofinder, and aligned using MUSCLE v3.8.31 (40). A maximum likelihood tree was constructed using IQ-Tree v1.6.12 (51) with the LG amino acid substitution model (the best fitting model according to ProtTest v3.4.2 (52)) with 1000 ultrafast bootstraps, and visualized using Figtree v1.4.4 with midpoint rooting.

Repeat Analysis

Repeat content was identified using Repeatmodeller v.2.0.1 (53) with rmblast v.2.10.0+ and Tandem Repeat Finder v.4.09 (54), RepeatScout v.1.06 and RepeatMasker v.4.0.5 (56). The output of Repeatmodeller (consensi.fa.classified) was then used as a library for RepeatMasker. The repeat content and family distribution for each chytrid species was determined from RepeatMasker.out, excluding lower scoring matches whose domain partly (<80%) includes the domain of another match.

TE and repeat distributions in the genome were assessed using gff files converted from the Repeatmasker .out files with a custom script that stringently excludes lower scoring matches whose domain partly (<80%) includes the domain of another match and visualized using IGV 2.8.2 (57). Repeatmaskers -GFF option does include those lower scoring, overlapping repeats and is therefore not suitable for further in depth repeat distribution analysis. TE distribution in relation to GC content was analyzed using Pilon's GC.wig files. Additionally, TE and repeat content of 10kb windows assigned to different quadrants was calculated using custom scripts based on RepeatMasker .out files and lists of genes assigned to quadrants (https://bitbucket.org/Theresa_42/wackeretal_2022_bsal_2speedgenome/).

To assess if repeat content was correlated to genome assembly quality, Spearman's Rank Correlation Coefficients, Spearman's correlation and linear regression (linear model fitting based on formula by Wilkinson and Rogers (1973) (58)) were calculated between repeat content and N_{50}, the number of contigs, genome length, as

well as the number of genes using ggpubr (https://github.com/kassambara/ggpubr). This was done for both all genome assemblies, as well as genome assemblies partitioned by sequencing technology.

For the heatmap showing repeat superfamily profiles, heatmap.2 was used with hierarchical clustering and Euclidian as a distance measure. Repeat families that did not exceed 1% abundance in any of the chytrids were excluded. Repeat families in *Bd* and *Bsal* were aligned using blastn with an e-value of 0.01, no filtering (-dust no, -soft_masking false) and a wordsize of 7 to determine homologs. Telomeric sequences were manually curated.

All resulting repeat consensus sequences were translated into the six possible reading frames of protein sequences using Emboss transseq (with -clean option (59)) and were scanned for Pfam HMM profiles (Pfam database release 35.0 (37, 60)) using hmmsearch (e-value < 1e-3 (61)) and for CDD profiles (version 3.19 (62)) using RPS-BLAST (e-value < 1e-3; with parameters -seg no -comp_based_stats (63)) and the results compared to a manually curated list of domains associated with TE activity or related to TEs based on published literature (**Dataset S2**, (3, 64–67)). LINEs were classified as fully functional and active if they contained any reverse transcriptase Pfam or CDD domain and an additional apurinic endonuclease domain and as autonomous if they contained a reverse transcriptase domain only, based on (3). LTRs were considered fully functional and active if they contained domains associated with a reverse transcriptase, a GAG capsid protein, a aspartic proteinase and an integrase and considered autonomous if they contained a reverse transcriptase, based on (3). Class II DNA transposons were considered active and autonomous if they were found to have domains associated with transposases. The same analysis was conducted on individual, non-consensus repeat sequences of BatrLINE-1, BatrLINE-2 and BatrLINE-3 found in *Bsal,* using all repeats identified by Repeatmasker.

Individual, non-consensus repeat sequences of BatrLINE-1 were aligned using MAFFT v7.490 (--localpair and –reorder options) (68), using all repeats. A maximum likelihood tree was constructed using IQ-Tree v1.6.12 (51) with GTR+F+R10 as a substitution model, determined using ModelFinder (69). Branch supports were obtained with the ultrafast bootstrap (n=1000) and branches with less than 30% support were

collapsed (-minsup 0.3) (70). The tree was visualized using iTOL v6 (43) with midpoint rooting.

Analysis of RIP activity and RNAi machinery

To assess if *Bsal*'s genome has evidence of repeat induced point mutations (RIP), all individual non-consensus entries for each repeat family were extracted from the genome and aligned using MAFFT v7.490 (--localpair and –reorder options) (68). Alignments were then tested for signatures of RIP activity using RIPcal (6) with the degenerate consensus sequence as reference, as well as dinucleotide frequencies of core conserved chytrid genes, aligned with MAFFT (--localpair and –reorder options).

Bsal's genome was scanned for RIP using RIPper v1.0 with default parameters (window size: 1000bp, sliding window step size: 500bp) (4). RIPper was used to automatically calculate the RIP substrate index $[(CpA + TpG)/(ApC + GpT)]$ (4, 6, 7), the RIP product index $[TpA/ApT]$ (4, 6, 7) and the RIP composite index $[(TpA/ApT) - ((CpA + TpG)/(ApC + GpT))]$ (4, 5), while average repeat family dinucleotide index values generated using RIPcal were used to calculate the RIP substrate and the RIP product index. Values of below 0.75 for the RIP substrate index were considered RIP affected (4, 7). Values of the RIP product index > 1.1 were considered RIP affected, while RIP product index values < 0.8 were indicative of the absence of RIP (4, 7). Negative values of the RIP composite index indicated an absence of RIP signatures (5).

RIP defective (RID) candidates in *Bsal*'s genome were identified using Interpro v87.0 (71) and Pfam (37) scan (Interpro domains: IPR018117, IPR003356, IPR001525; Pfam domains: PF00145) as described before (72). Three candidate C-5 cytosine methyltransferases were identified: BSLG_000286, BSLG_000626 and BSLG_010741. They were aligned to 15 known RID proteins (**Dataset S3**) using MAFFT as described (72) before and screened for an amino acid change of NV to QT or ET in motif VI and visualized in Jalview 2.11.2.5 (73). All three putative RID C-5 cytosine methyltransferases did not have an amino acid change in motif VI (**Figure S11**). Additionally, homologs of 15 known RID proteins were searched for using blastp (e-value < $1e^{-5}$) (63). No homologs were identified.

The RNAi machinery in all chytrids was identified by screening all genomes for homologs to representative Dicer, Argonaute/piwi (Ago), and RNA dependent RNA polymerase (RdRP) proteins, listed in **Dataset S3** using Blastp and tBlastn (e-value < $1e^{-5}$) (63, 74).

Genome compartmentalization analysis

Flanking intergenic distance was calculated for all non-terminal protein coding genes based on gff3 files. For each chytrid species, density plots of intergenic distances were constructed for all non-terminal protein coding genes, and several gene subsets including genes with a secretion signal, SSPs, conserved chytrid BUSCO genes, differentially expressed genes and M36 metalloproteases. The median 5' and 3' intergenic distance for all protein coding genes in a given species was used to define four quadrants including bottom left (gene-rich/repeat-sparse; Q_{SS}), top right (gene-poor/repeat-rich; Q_{LL}), bottom right (long 3' intergenic distance, short 5' intergenic distance; Q_{LS}) and top left (short 3' intergenic distance, long 5' intergenic distance; Q_{SL}).

To identify enrichment of gene categories in each quadrant, hypergeometric tests were performed on all genes, the aforementioned four gene categories, and the largest 10 secreted families (determined by MCL; https://bitbucket.org/Theresa_42/wackeretal_2022_bsal_2speedgenome/). Hypergeometric tests were also used to determine enrichment for flanking repeat families and to determine whether genes falling in one of the quadrants are enriched on certain chromosomes compared to the overall distribution of genes in quadrants on all chromosomes. Critical *p*-values for hypergeometric and X^2 enrichments were determined using Bonferroni correction with an α-level of 0.01. For gene category enrichment, we performed 16 tests = 0.00063. For secreted families and quadrant enrichments tests on chromosomes, we performed 4 tests = 0.0025. For flanking repeat families, the correction was adjusted based on the total number of repeat families flanking.

There is currently no known population structure of *Bsal*, thereby precluding the study of intra-population genetic variation. However, there are multiple lineages of *Bd* described (75, 76) (albeit only *Bd*GPL has high quality annotated genome assemblies) and therefore genetic variation for this species can be compared to intergenic distance. Paired-end Illumina data from representatives of all five known lineages (*Bd*GPL

JEL423, *Bd*CAPE TF5a1, *Bd*CH ACON, *Bd*Asia-1 KRBOOR_323, *Bd*Asia-2 CLFT065, and a hybrid of unknown parentage SA-EC3) were obtained from the NCBI Sequence Read Archive (SRA) (75–77). The Genome Analysis Toolkit (GATK) v.4.1.2.0 (78) was used to call variants. Our Workflow Description Language (WDL) scripts were executed by Cromwell workflow execution engine v.48 (79). Briefly, raw sequences were pre-processed by mapping reads to the reference genome *Bd* JEL423 using BWA-MEM v.0.7.17 (80). Next, duplicates were marked, and the resulting file was sorted by coordinate order. Intervals were created using a custom bash script allowing parallel analysis of large batches of genomics data. Using the scatter-gather approach, HaplotypeCaller was executed in GVCF mode with the diploid ploidy flag. Variants were imported to GATK 4 GenomicsDB and hard filtered (QD < 2.0, FS > 60.0, MQ < 40.0, GQ ≥ 50, AD ≥ 0.8, DP ≥ 10).

The direction and magnitude of natural selection for each lineage was assessed by measuring the rates of non-synonymous substitution (*dN*), synonymous substitution (*dS*) and omega ($\omega = dN/dS$) using the yn00 program of PAML (81) implementing the Yang and Nielsen method, taking into account codon bias (82). The program was run on every gene in each isolate using the standard nuclear code translation table. Hypergeometic tests were calculated for genes with $\omega > 1$ in each quadrant. We performed 20 tests per lineage; thus, the *p*-value was Bonferroni corrected to 0.0005 at an α-level of 0.01

X^2 enrichments tests of independence were performed on a range of genes including 1) genes with a secretion signal, 2) SSPs, 3) conserved chytrid BUSCO genes, 4) repeat families flanking genes coding for secreted proteins, 5) M36 metalloproteases 6) differentially expressed genes and 7) *Bd* genes that have $\omega > 1$. Briefly, 2x2 contingency tables were generated for each test, comprising two groups of dichotomous variables (number of genes in or not in the gene category of interest, and the number of genes in or not in a given quadrant). X^2- tests for goodness of fit were performed to determine whether the distribution of genes within each quadrant was significantly different from the expected distribution (25% each). X^2- tests were performed on each contig iteratively to test for genomic hot-spots for rapid evolution.

Wilcoxon rank-sum tests were computed to test the null hypothesis that the log_{10} mean intergenic distances of the feature category of interest (SSPs, HKGs, M36 metalloproteases and secreted proteins only) and the log_{10} mean intergenic distances of all the other genes that are either a) not in that feature category of interest or b) of a different feature category have the same continuous distribution (https://bitbucket.org/Theresa_42/wackeretal_2022_bsal_2speedgenome/). Wilcoxon rank-sum tests were performed using Rstatix v0.7 (https://cran.r-project.org/web/packages/rstatix/index.html) wilcox_test (conf.level=0.95). Wilcoxon effectsize was determined using Rstatix wilcox_effsize (conf.level=0.95,nboot=1000 and ci=TRUE). For Wilcoxon rank sum tests, the adjusted p-values in the violin plots for α-levels of 0.01 to 0.0001 and 6 tests were as follows: ns: $p > 0.0017$, *: $p \leq 0.0017$, **: $p \leq 1.7E-4$, ***: $p \leq 1.7E-5$, ****: $p \leq 1.7E-6$.

Consecutive gene counts were generated using lists of genes assigned to their quadrants as defined above and a bespoke script. To assess the significance of finding a sequence (*n*) of any given length (*k*) of consecutive genes of the same quadrant, a discrete pattern Markov chains were used. The probability of transitioning from one quadrant to the next was set to 0.25. Based on that, a (k+1)(k+1) transition matrix was generated. Once the transition matrix was constructed, for a given value of n the probability of having the number of consecutive genes of a certain quadrant in the chain was $P(W|n) = \{Pn\}0_{,k}$. In the calculation, n was set to 100 repetitions of equiprobable outcome. W is the event of the occurrence of k consecutive genes of the same quadrant.

Differential expression analysis

To analyse gene expression, we obtained our *Bsal in vitro* RNAseq reads (NCBI BioProject PRJNA326249), *Bd in vitro* RNAseq reads (PRJNA326253), and *Bd* or *Bsal* infected *Tylototriton wenxianensis* (PRJNA300849). Full details of the animal experiment and RNA extraction are provided in the “Host–pathogen transcriptomes” section of the Methodology in the corresponding publication: (2). Briefly, nine captive bred *Tw* were housed individually at 15C. All animals were clinically healthy and free of *Bd* and *Bsal* as assessed by sampling the skin using cotton tipped swabs and subsequent performing qPCR. After 1 week of acclimatization, six animals were exposed to 1ml of 104 *Bd* (three animals) or *Bsal* (three animals) spores per ml water for 24 h6. Three additional animals

were used as negative controls and were sham treated with 1ml water for 24 h At 10 days post infection (*Bd* infection load 1,100 GE per PCR reaction; *Bsal* infection load 1,900 GE per PCR reaction) the animals were killed and the skin was removed immediately. A part of the skin (10 mg) was stored in RNA later for 24 h and RNA then extracted with TRI Reagent combined with the RNeasy Plant kit (Qiagen). Strand-specific libraries were constructed for each of the three replicates for each condition, using TruSeq RNA sample preparation with poly-A selection, and paired-end reads were generated on an Illumina HiSeq2000.

Replicates for *Bd* and *Bsal* RNAseq (either *in vitro* or *ex vivo*) were compared separately using the Trinity v2.13.2 differential expression pipeline (83). The pipeline first aligns the reads to the coding sequences of *Bd* or *Bsal,* as well as the consensus repeats of *Bsal*, using Bowtie2 version 2.3.4.3 (84) (parameters --no-mixed --no-discordant --gbar 1000 --end-to-end -k 200), and then processed using Samtools version 1.8 (25) (using htslib 1.8, commands: view -F 4 -S -b | samtools sort -n -o). Transcript abundances was estimated using RSEM version 1.3 (85). Differential expression was predicted with our replicates using EdgeR (86), with significance set at FDR *p* value < 0.001 and > 4 fourfold change of TMM normalized FPKM.

The genetic distance between amphibians and fungi results in very low levels of cross-kingdom false positive alignments during the differential expression pipeline (i.e., amphibian RNA aligning to chytrid genes or vice versa). To experimentally check this, we aligned all of the RNAseq to the annotated genome assembly of the North American Bullfrog *Lithobates catesbeianus* (*Lc)* (NCBI genome accession GCA_002284835.2) (*Tylototriton wenxianensis* does not have a genome assembly, and any genomic region that is conserved between *Tw* and chytrids is likely to also be conserved between *Tw* and *Lc*) (**Table S2**).

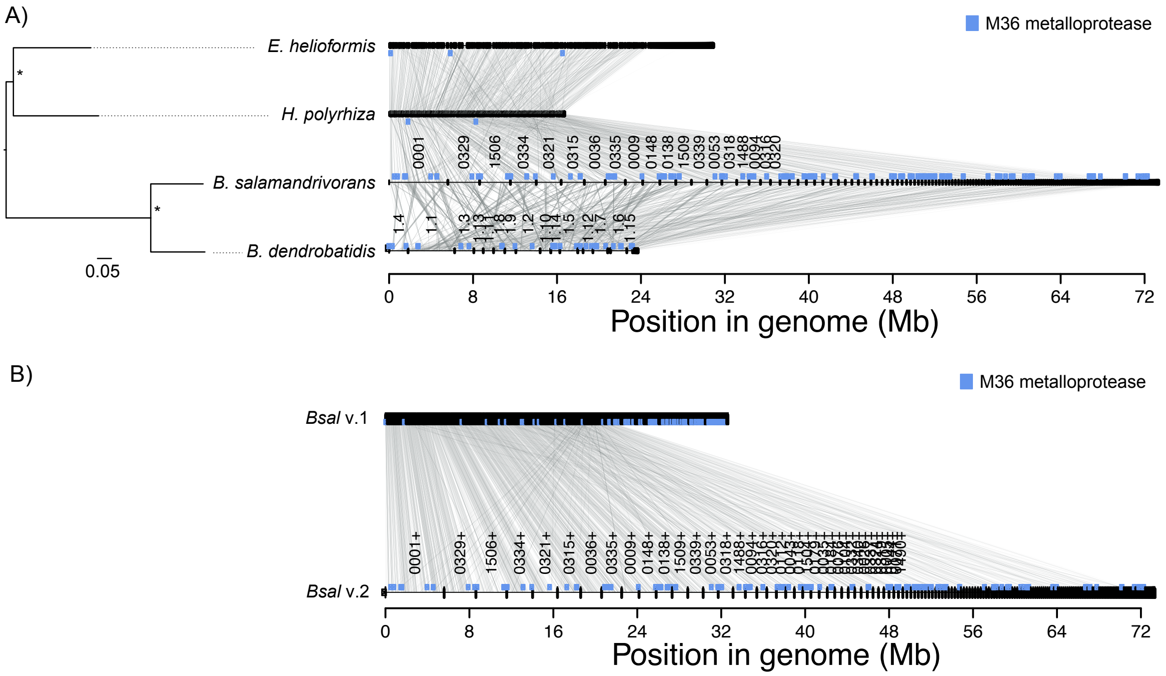

Figure S1. Synteny plots between **A)** *Bsal* and its three closest relative *Bd*, *Hp* and *Eh*, and **B)** *Bsal* v.1 genome assembly and *Bsal* v.2 genome assembly. The positions of M36 metalloproteases are indicated as blue squares. The phylogenetic tree of *Bsal* and its three closest relatives was constructed using core-ortholog multiple alignment and RAxML (with WAG transition matrix). Branch lengths indicating the mean number of nucleotide substitutions per site. Asterisks indicate 100% bootstrap support from 1,000 replicates.

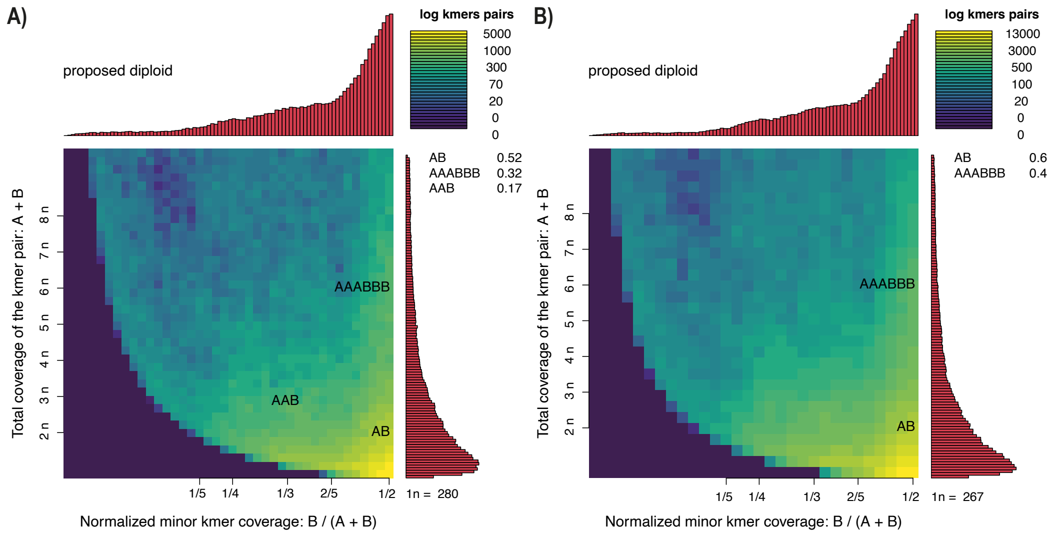

Figure S2. Smudgeplots of *Bsal* sequencing reads with kmer size A) 21 and B) 30.

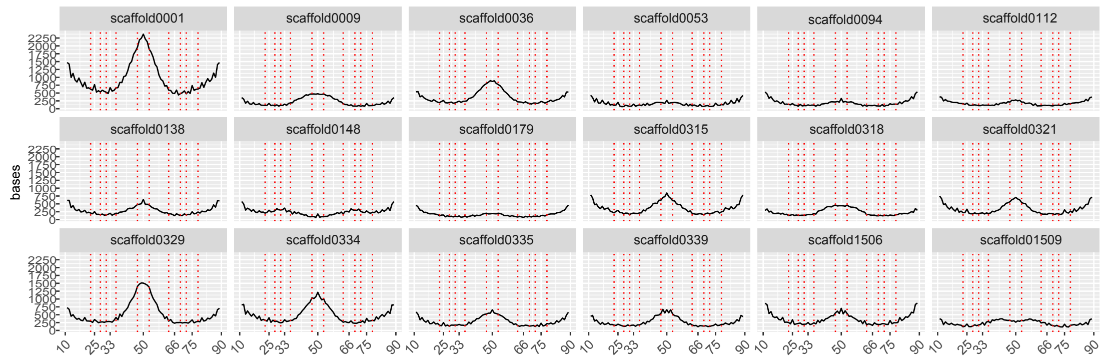

Figure S3. Allele frequencies for representative chromosomes of *Bsal*'s genome assembly. The x-axis represents the percent of reads specifying most frequent alleles.

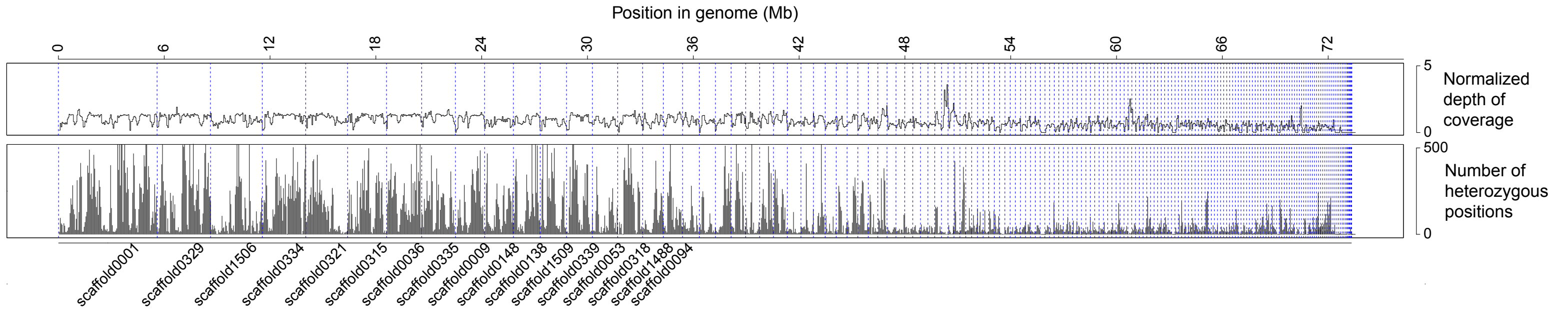

Figure S4. (top) Normalized depth of coverage of Illumina reads aligned to *Bsal*'s genome assembly, summarised using non-overlapping 50 kb windows. (**bottom**) Number of heterozygous positions found in non-overlapping 50 kb windows.

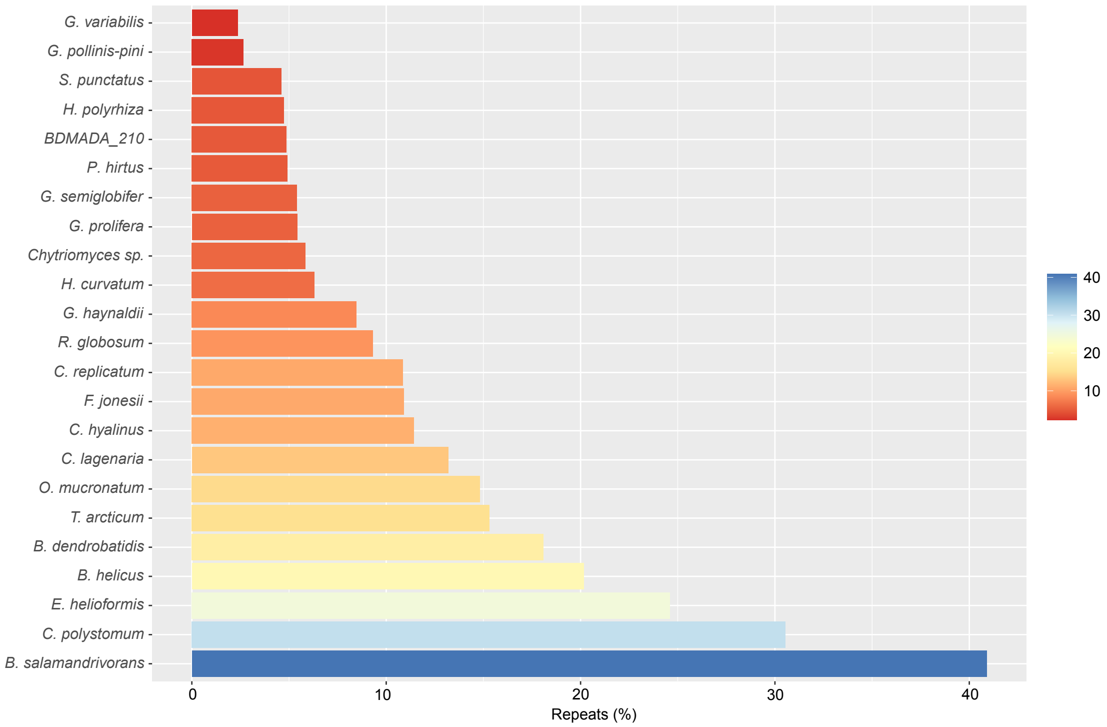

Figure S5. Overall repeat content (%) of all 22 chytrids. Overall repeat content in percent of all 22 chytrids excluding lower-scoring matches.

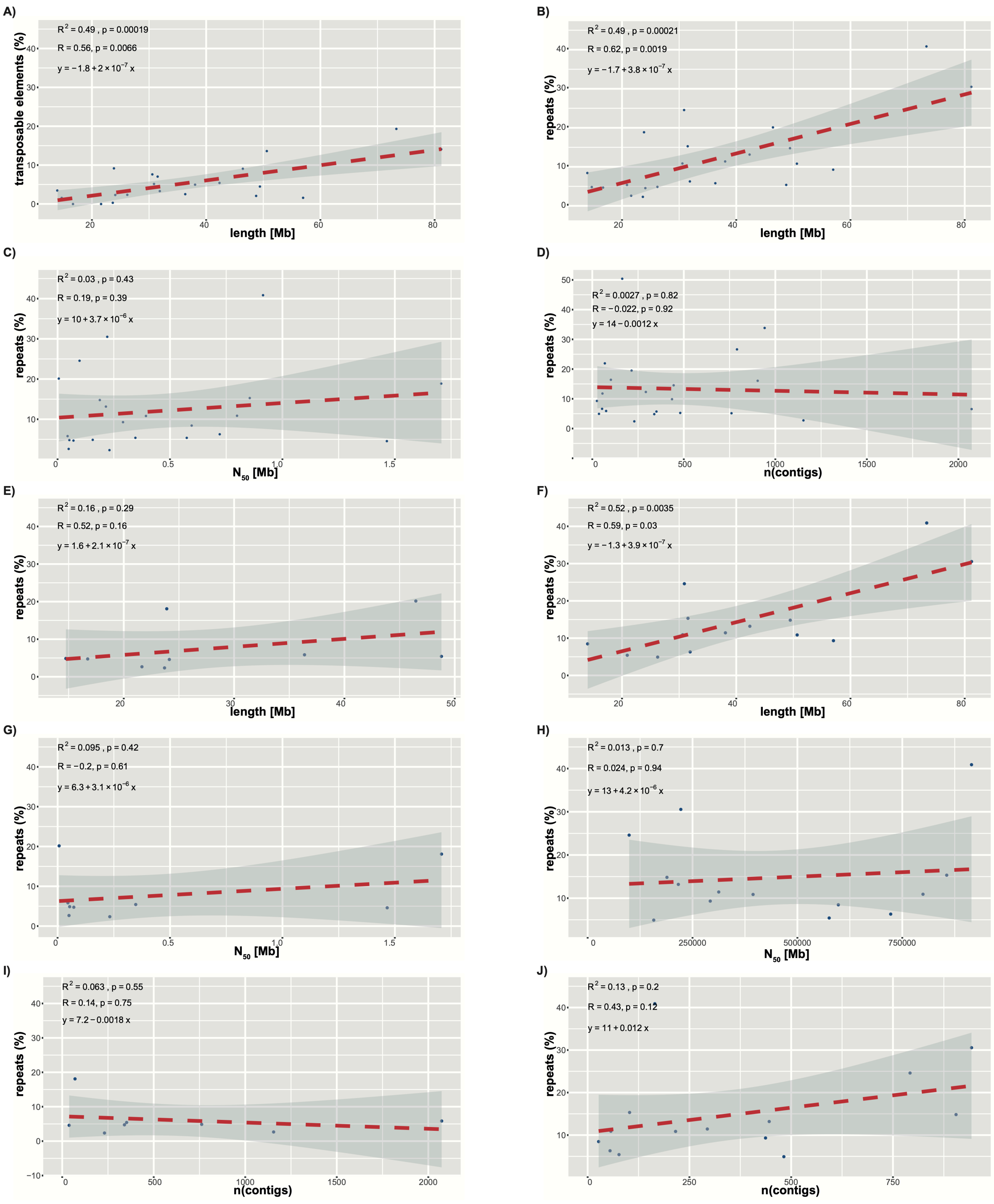

Figure S6. Linear regression plots with Spearman correlation coefficients. **A)** Correlation plot of transposable element content (%) and genome length (bp) for 22 chytrids, **B)** repeat content (%) and the genome length, **C)** repeat content (%) and N_{50} (%) and **D)** repeat content and the number of contigs, **E)** repeat content (%) and genome length for short read sequenced genomes (SRS) only, **F)** repeat content (%) and genome length for long read sequenced genomes (LRS) only, **G)** repeat content (%) and N_{50} for SRS only, **H)** repeat content (%) and N_{50} for LRS only, **I)** repeat content (%) and number of contigs for SRS only and **J)** repeat content (%) and number of contigs for LRS only.

Figure S6 *continued.* R-squared (R^2), equation of the linear equation and Spearman's rank correlation coefficient are indicated in each plot. Data points are blue, the linear regression line is red, the confidence interval is grey.

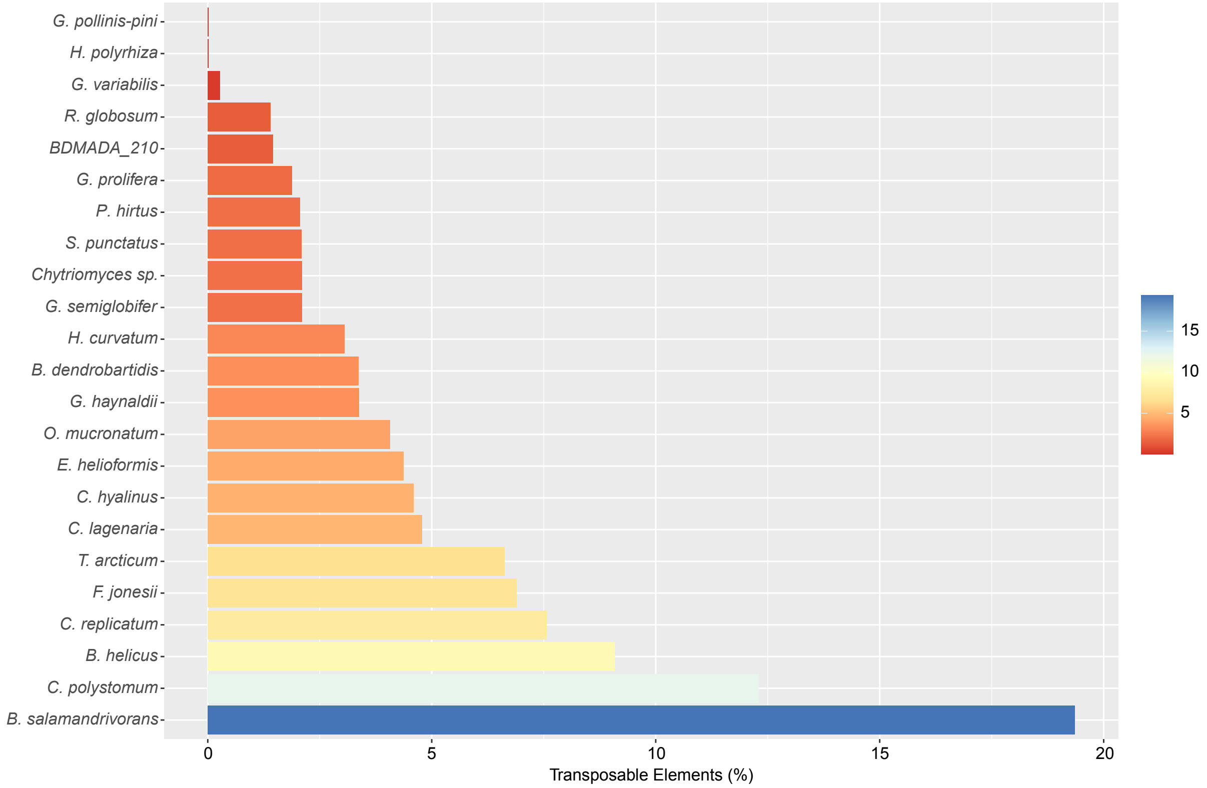

Figure S7. Overall content of transposable elements (%) of all 22 chytrids. Overall content of transposable elements in percent of all 22 chytrids excluding lower-scoring matches (stringent criterion).

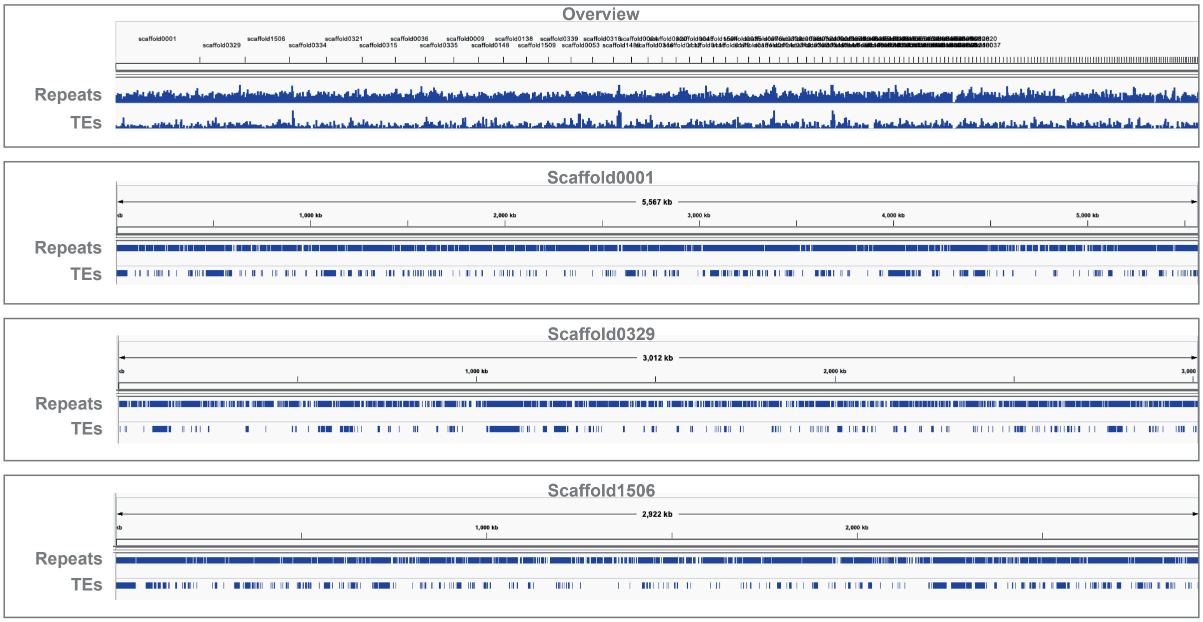

Figure S8. Repeat and TE distributions in the *Bsal* genome.

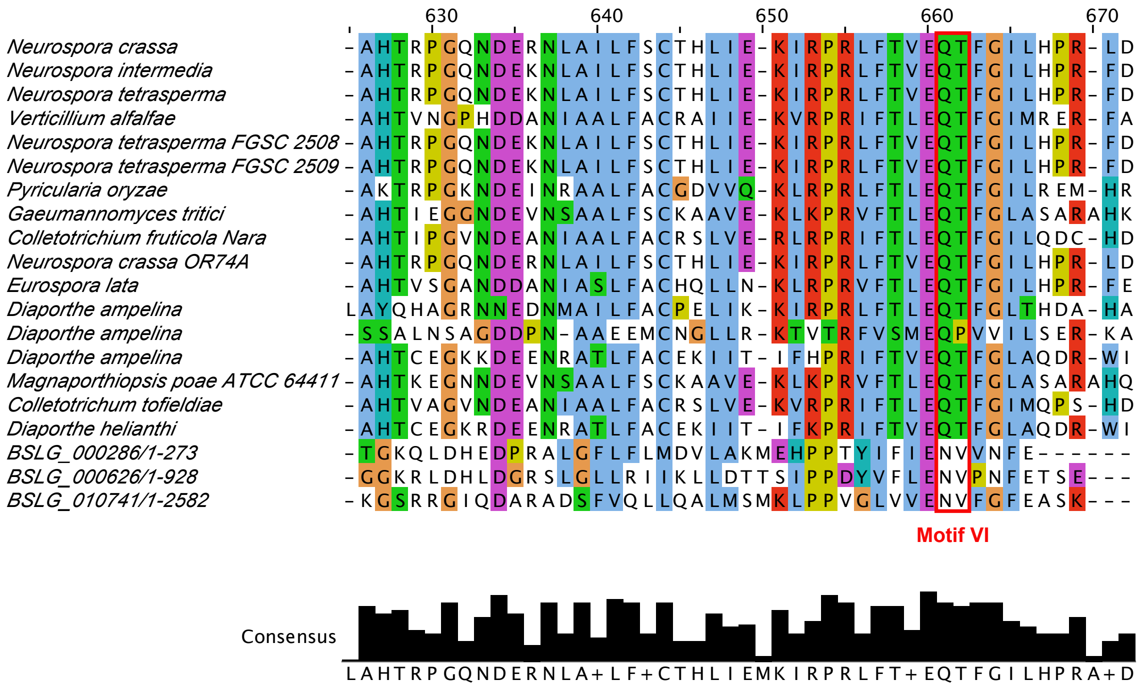

Figure S9. MAFFT alignment of known RID (RIP defective) sequences of fungi to putative C-5 cytosine methyltransferases of *B. salamandrivorans* (BSLG). C-5 cytosine methyltransferases with RID activity have a characteristic amino acid change from NV to ET or QT in motif VI; this change is not found in *Bsal*'s putative C-5 cytosine methyltransferases, confirming they are not RID.

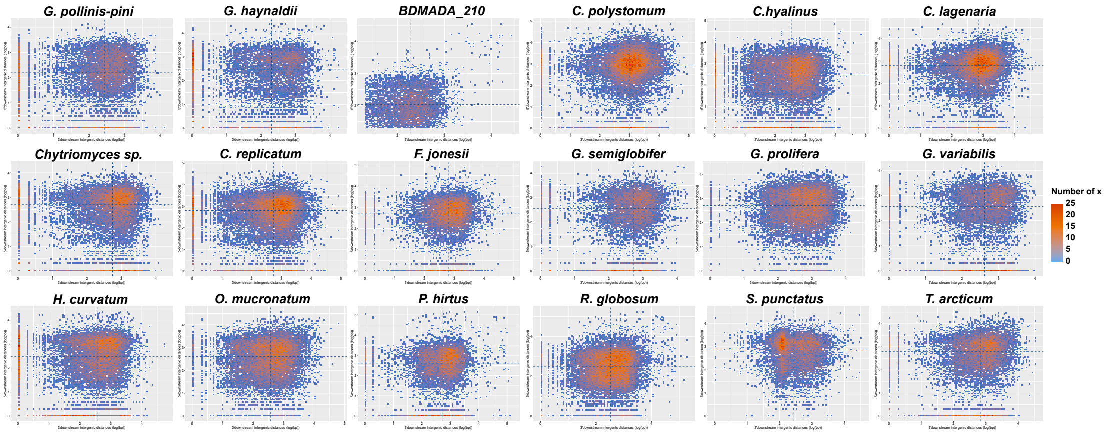

Figure S10. Density plots for all 22 chytrids analyzed. Density plots of intergenic distances for all non-terminal protein coding genes are shown (measured as $\log_{10}$ length of 5' and 3' flanking upstream and downstream intergenic distances), with the median values shown in dotted blue line.

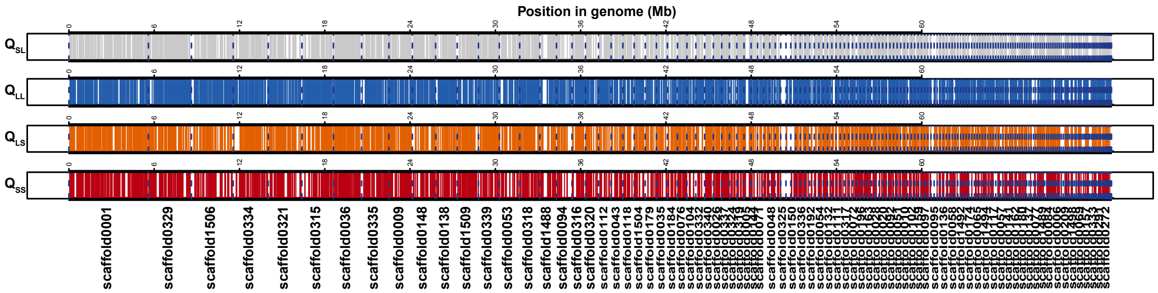

Figure S11. Distribution of 1kb windows designated to one of the four quadrants in the genome.

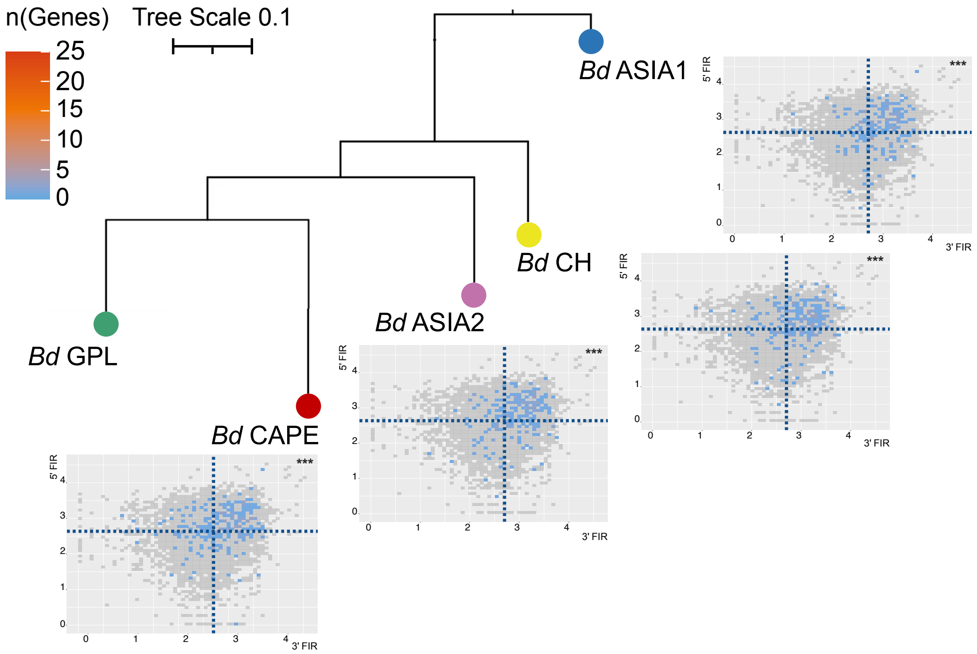

Figure S12. Density plots for *B. dendrobatidis* lineages for genes with a signature of positive selection (ω > 1). Density plots of intergenic distance for all non-terminal protein coding genes with signatures of positive selection (*dN*/*dS* (ω) > 1) are shown (measured as $\log_{10}$ length of 5' and 3' flanking intergenic distances; FIRs), with the median values shown in dotted blue line. Phylogenetic tree based on *Sewell et al.* (87). Vertical branch lengths indicating the mean number of nucleotide substitutions per site.

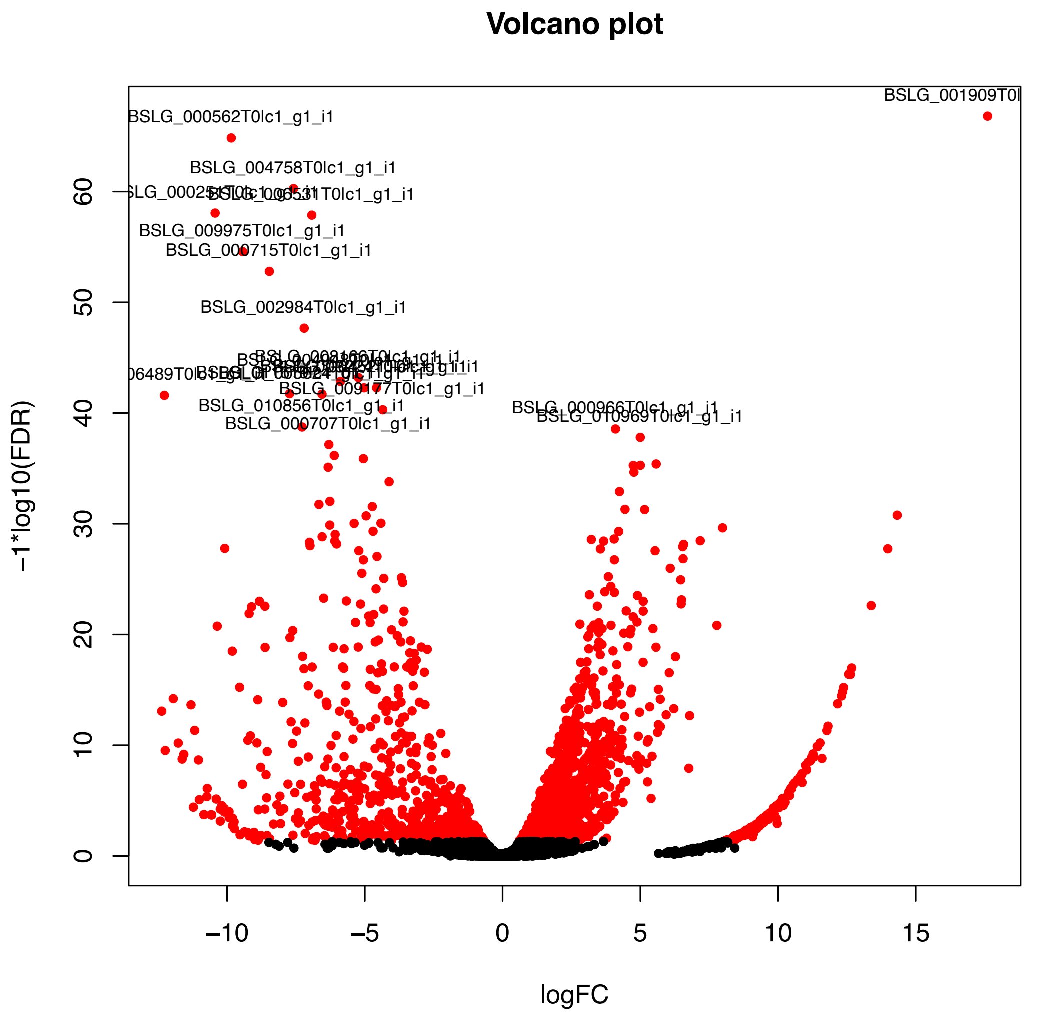

Figure S13. Visualization of *Bsal* DEGs volcano plot. The x-axis shows the log-fold change (logFC), the y-axis, -$\log_{10}$ of a *p*-value. The *p*-values < 0.05 are in red dots. Black dots indicate the remaining genes present which showed no significant change

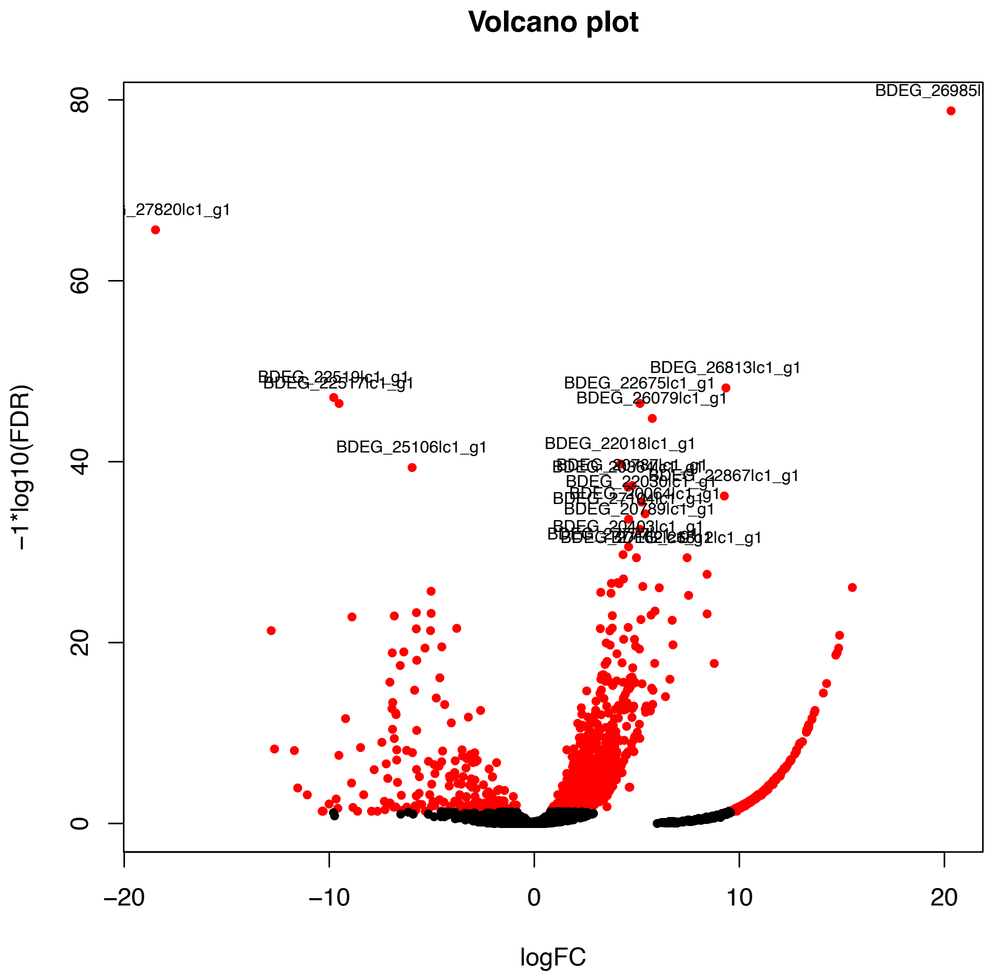

Figure S14. Visualization of *Bd* DEGs volcano plot. The x-axis shows the log-fold change (logFC), the y-axis, -$\log_{10}$ of a *p*-value. The *p*-values < 0.05 are in red dots. Black dots indicate the remaining genes present which showed no significant change.

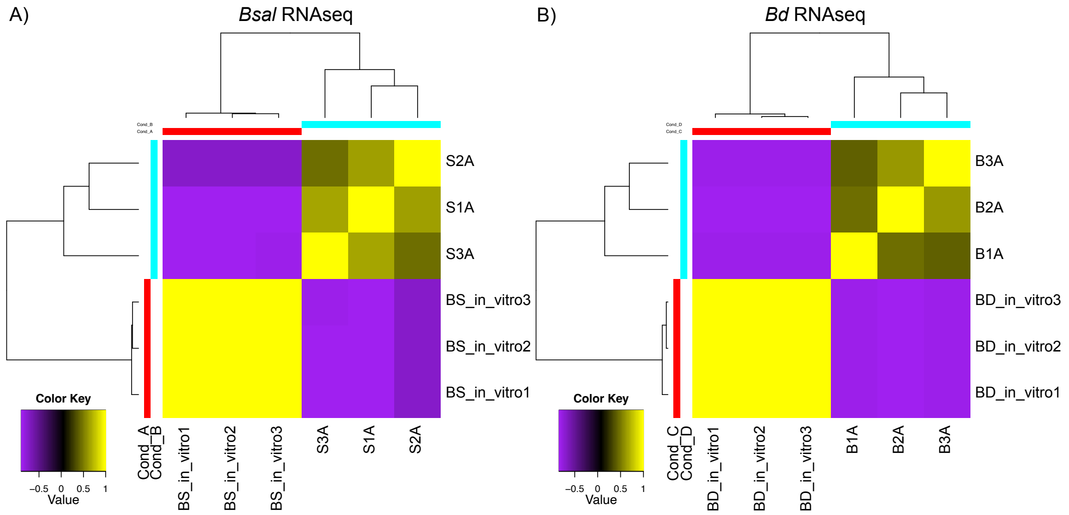

Figure S15. The correlation of expression values between replicates and conditions of *Bsal* (A) and *Bd* (B). Trees indicate hierarchical clustering of samples and replicates. Cond_A = *Bsal in vitro*; Cond_B = *Bsal in vivo*; Cond_C = *Bd in vitro*; Cond_D = *Bd in vivo*; S1-3A = *Bsal in vivo* samples; B1-3A = *Bd in vivo* samples. Correlation values indicated in Color Key are Pearsons correlation coefficient values.

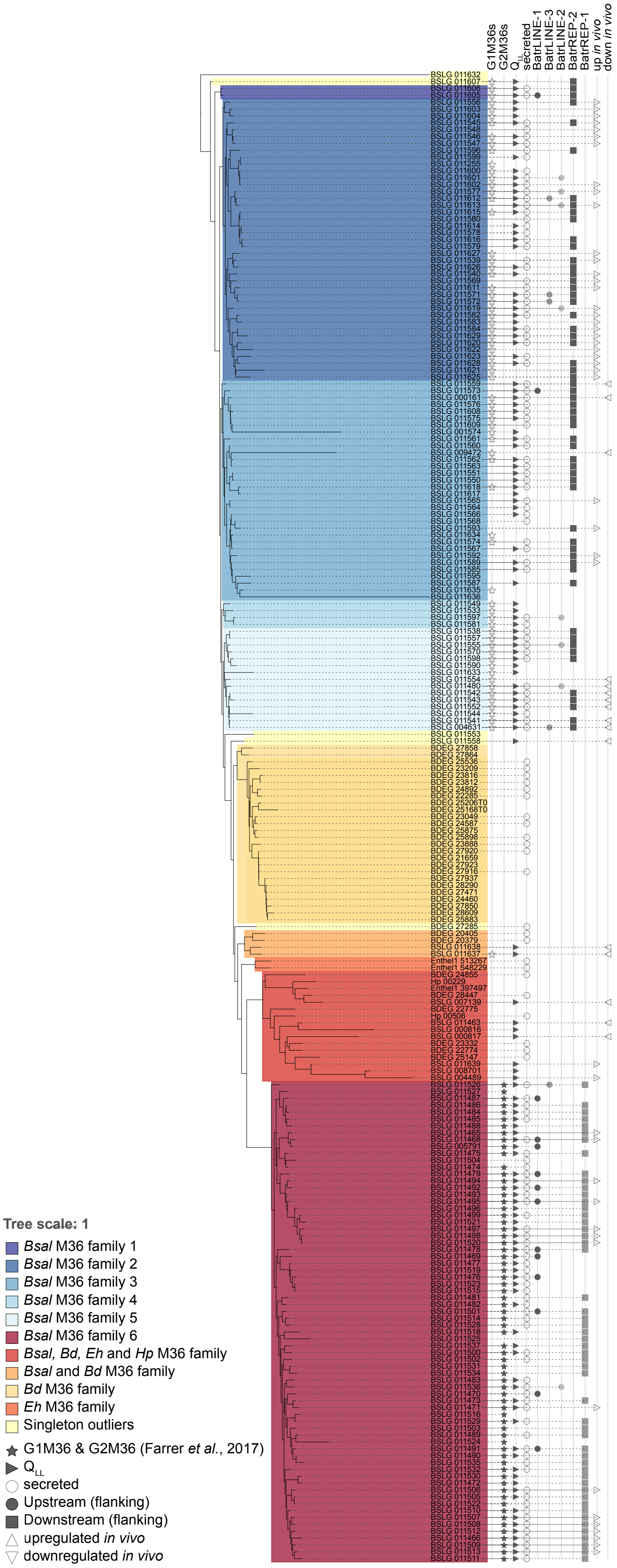
G1M36s
G2M36s
Q_{LL}
secreted
BatrLINE-1
BatrLINE-3
BatrLINE-2
BatrREP-2
BatrREP-1
up *in vivo*
down *in vivo*
Tree scale: 1
Bsal M36 family 1
Bsal M36 family 2
Bsal M36 family 3
Bsal M36 family 4
Bsal M36 family 5
Bsal M36 family 6
Bsal, *Bd*, *Eh* and *Hp* M36 family
Bsal and *Bd* M36 family
Bd M36 family
Eh M36 family
Singleton outliers
G1M36 & G2M36 (Farrer *et al.*, 2017)
Q_{LL}
secreted
Upstream (flanking)
Downstream (flanking)
upregulated *in vivo*
downregulated *in vivo*

Figure S16. A gene tree inferred using RaxML from protein alignments of all identified M36 proteins in four chytrids (*Bsal*, *Bd*, *Eh* and *Hp*). The branch lengths (Tree scale) indicate the mean number of nucleotide substitutions per site. Presence of 5 upstream and downstream flanking repeat families (BatrLINE-1, BatrLINE-2 and BatrLINE-3, BatrREP-1 and BatrREP-2) are indicated as circles (BatrLINEs) and squares (BatrREPs). Previous M36 family characterizations (G1M36 and G2M36) (2) are marked by star outlines and filled star shapes, respectively. Genes falling into the gene-sparse region Q_{LL} are indicated by a filled triangle shape. Secreted genes are denoted by an open circle, genes up and down regulated *in vivo* point are designated big open triangles pointing right or left, respectively. LINEs with a functional reverse transcriptase (RT) domain are marked in dark red. Gene IDs: Enthel1 = *E. helioformis*, *Hp* = *H. polyrhiza*, BSLG = *B. salamandrivorans* and BDEG = *B. dendrobatidis.*

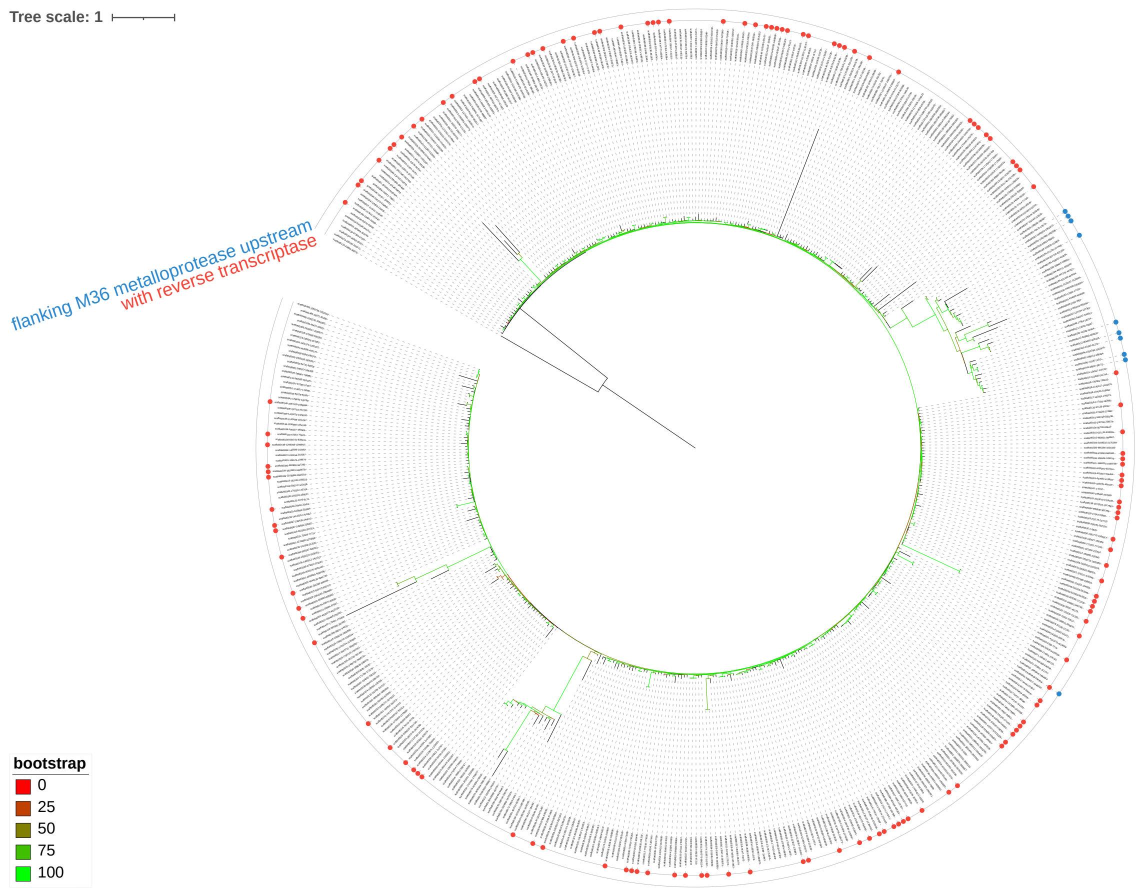

Figure S17. Maximum likelihood tree inferred from MAFFT alignments of individual LINE BatrLINE-1 sequences. Branch supports were obtained with the ultrafast bootstrap (*n* = 1000) and branches with less than 30% support were collapsed. Blue circles mark BatrLINE-1 sequences that are found upstream of M36 metalloproteases. Red circles denote BatrLINE-1 sequences that have a detectable reverse transcriptase (RT) domain.

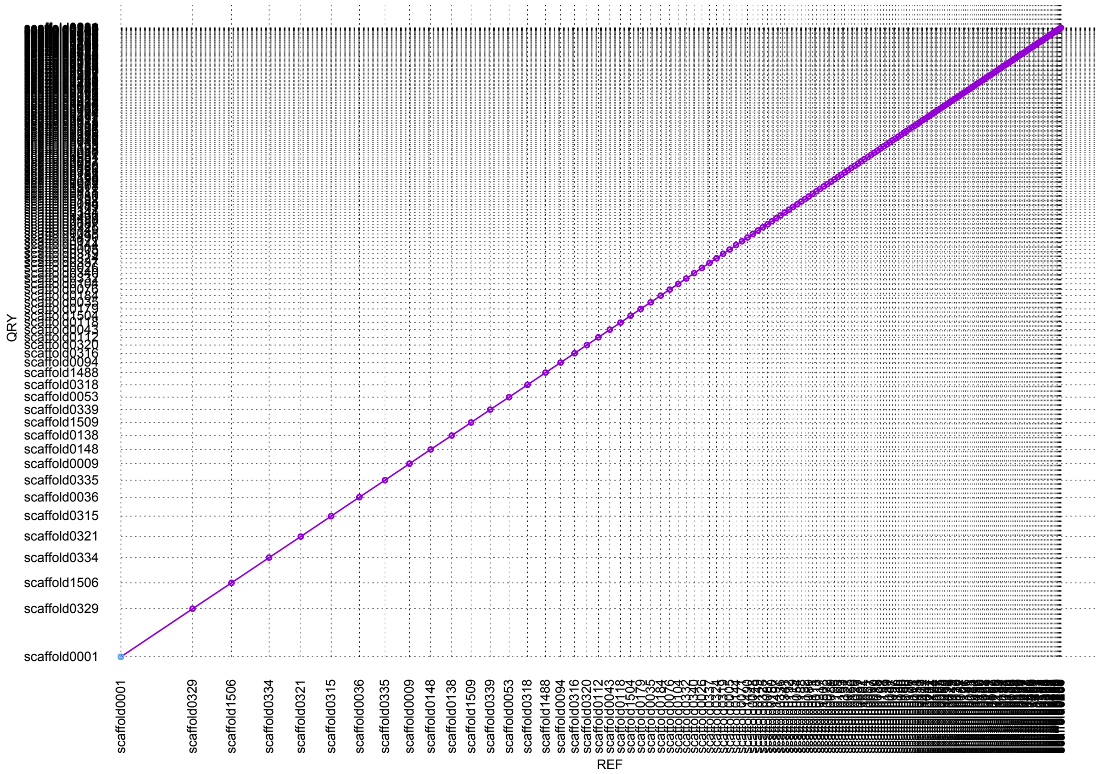

Figure S18. Dot plot (MUMmer plot) of the alignment of the masked *BsaI* genome assembly *vs.* itself using MUMmer (mummerplot parameters: --layout and --filter; nucmer parameter: --nosimplify).

Table S1. Whole genome assembly statistics. All genome assemblies apart from V1 Allpaths (2), are *de novo* long read assemblies generated in this study. V2 Canu default settings Pilon corrected = *Bsal* assembly v2.0 (our chosen assembly, highlighted in blue).

	Parameter	V1 Allpaths	V2 Cau default settings	V2 Canu default setting and Pilon corrected	V2 Canu default settings and Medaka polished	V2 Canu default Medaka Pilon corrected	V2 Canu default Medaka Pilon Reapr	V2 Canu non-default settings	V2 Raven default settings
Quast	no. of contigs	5358	165	165	174	174	694	179	190
	total length [bp]	32636440	73003593	73346731	73767267	73659790	76312616	75723379	78607535
	largest contig [bp]	68925	5565889	5593528	5635152	5621833	5621833	4327542	1817668
	N50	10956	910925	915035	920098	919980	831444	1480457	561050
	GC [%]	41.89	42.86	42.93	43	43.01	43.16	42.87	41.8
Coverage	mean normalized coverage	0.42	0.82	0.83	0.83	0.84	N/A	0.71	0.83
	σ normalized coverage (50 kb w.l.)	0.82	0.43	0.43	0.39	0.39	N/A	0.66	0.78
	Maximum normalised coverage (50 kb w.l.)	13.2	3.40	3.59	4.91	4.66	N/A	14.98	13.95
Reapr	error free bases [%]	84.82	50.33	66.71	65.79	68.33	66.62	39.66	51.01
	no. of gaps	452	0	0	0	0	10634	0	0
	total gap length [bp]	173212	0	0	0	0	5691950	0	0
	FCD errors within contig	1890	13855	14238	7236	7787	751	13494	8980
	FCD errors over gap	86	0	0	0	0	731	0	0
	low fragm. cov. within a contig	1180	39323	29475	12473	11624	2756	42192	15182
	low fragm. cov. over a gap	26	0	0	0	0	876	0	0
	collapsed repeats	1386	2910	2421	902	836	760	25024	1175
	low perfect cov.	6959	90107	39444	38671	28938	23805	92728	43567
	low read cov.	6	10993	10636	3173	3094	2552	10582	16896
	wrong read orientation	7054	41762	26684	11860	9988	16254	42223	20424
	new no. of contigs	5471	1676	1766	709	694	2753	1706	1699
	new total length [bp]	32625002	77758845	78366536	76446839	76312616	76962701	80666216	100287279
	new N50	10846	788500	793347	831837	831444	139969	1007928	409982
	new largest contig [bp]	68925	5565889	5593528	5635152	5621833	745844	4327542	1817668
Mummer dnadiff	no. of gaps	2	3	0	10	29	N/A	8	19
	no. of duplications	270 (44 in masked)	1220	760 (0 in masked)	1044	2089	N/A	1608	1496
Duplication Analysis	No. of duplications	0	0	0	76	74	N/A	2	6

Table S2. RNAseq from the Farrer *et al.* (2) paper was aligned to the annotated genome assembly of the North American Bullfrog *Lithobates catesbeianus* to determine cross-kingdom false positive alignments.

RNAseq dataset	Total nt	Nt aligned	% nt aligned
Tylototriton wenxianensis	48626454242	2889053490	5.94
Bd infecting *Tw*	42599142892	2448359180	5.75
Bsal infecting *Tw*	47478190494	3176057110	6.69
Bd in vitro	15996870860	70908868	0.44
Bsal in vitro	14441379354	59894010	0.41

Table S3. Only 17 of the 22,238 annotated *Lithobates catesbeianus* genes had any coverage (breadth of coverage across the genome = BOC, depth of coverage = DOC) from the *Bd in vitro* and *Bsal in vitro* RNAseq datasets.

Gene Name	Gene Size	Bd BOC (%)	Bd DOC	Bsal BOC (%)	Bsal DOC
AB205_0219920	2767	24.83	3.72	17.28	3.98
AB205_0103770	2590	24.63	124.67	33.63	67.94
AB205_0114620	1791	17.20	2.53	20.99	0.16
AB205_0047760	2639	6.40	0.06	8.98	0.19
AB205_0169950	5712	5.36	0.02	5.57	0.06
AB205_0103780	6386	5.09	0.17	5.65	0.05
AB205_0009140	6795	1.71	0.02	0.00	0.00
AB205_0151560	8417	1.46	0.01	0.00	0.00
AB205_0149070	11864	1.03	0.01	0.00	0.00
AB205_0103370	12721	0.89	0.03	0.00	0.00
AB205_0213990	16539	0.70	0.01	0.00	0.00
AB205_0006310	19351	0.52	0.00	0.00	0.00
AB205_0108150	11736	0.27	0.00	0.00	0.00
AB205_0214420	1808	0.00	0.00	21.29	1.30
AB205_0016620	8557	0.00	0.00	3.10	0.04
AB205_0102160	8921	0.00	0.00	2.26	0.00
AB205_0143890	11928	0.00	0.00	1.09	0.05

Table S4. *Lithobates catesbeianus* genes with *Bd in vitro* or *Bsal in vitro* RNAseq aligning to them were BLASTp (or BLASTn for AB205_0169950) to the NCBI nr database, revealing highly conserved genes.

Gene	Top HSP with gene description (exc. Hyothetical)	E-value
AB205_0219920	Tubulin alpha-1A chain [Labeo rohita]	0
AB205_0103770	tubulin alpha 1a [Myotis myotis]	0
AB205_0114620	actin, cytoplasmic 2 [Geospiza fortis]	0
AB205_0047760	tubulin alpha-1B chain isoform X6 [Colobus angolensis palliatus]	0
AB205_0169950	Rana temporaria actin gamma 1 (ACTG1)	0
AB205_0103780	tubulin alpha-1B chain isoform X6 [Colobus angolensis palliatus]	0
AB205_0009140	tubulin beta-3 chain [Ictalurus punctatus]	0
AB205_0151560	homeobox protein Hox-B3 [Rana temporaria]	0
AB205_0149070	interleukin-17A-like [Rana temporaria]	2.00E-37
AB205_0103370	dynamin-binding protein isoform X1 [Rana temporaria]	0
AB205_0213990	tyrosine-protein kinase ZAP-70 [Rana temporaria]	5.00E-147
AB205_0006310	eukaryotic translation initiation factor 4 gamma 1 isoform X2 [Rana temporaria]	0
AB205_0108150	pseudouridylate synthase 7 homolog isoform X1 [Rana temporaria]	1.00E-120
AB205_0214420	elongation factor 1-alpha, somatic form [Rana temporaria]	0
AB205_0016620	tubulin beta chain [Xenopus tropicalis]	0
AB205_0102160	heat shock protein family A (Hsp70) member 8 L homeolog isoform X1 [Xenopus laevis]	0
AB205_0143890	calcium homeostasis endoplasmic reticulum protein [Rana temporaria]	9.00E-75

Table S5. Reads aligned to consensus repeat families of *Bsal*. Number and fraction (%) of repeat families, excluding simple repeats and low complexity repeats, that have reads from the *Bsal in vitro* and *in vivo* RNAseq datasets **(2)** aligned to them.

	in vitro	***in vivo***
**Repeatfamilies with aligned reads total (n)**	559	430
**Fraction of repeatfamilies with aligned reads (%)**	76.16	58.58
**Repeatfamilies with aligned reads in more than 2 samples(n)**	512	305
**Fraction of repeatfamilies with aligned reads in more than 2 samples (%)**	69.75	41.55

Dataset S1. Repeat superfamily distributions of the 22 chytrids based on RepeatModeller classifications. Lower-scoring matches were excluded. All values are in base pairs (bp). **A)** Total for each repeat. **B)** Summary.

Dataset S2. Conserved Domains Database (CDD) and Protein Family (Pfam) scan of repeat families in *Bd* and *Bsal*.

A) Pfam and CDD domains associated with transposable elements (TE) or TE activity based on and expanded from *Muszewska et al.* (65), with additional domains from LTRpred (66) and *Naish et al.* (67). TE superfamily, database (Pfam 35.0 or CDD 3.19), domain name assigned by database for entry, redundancy term for sorting of domains into common categories and domain ID are indicated. TRANS = transposase; DNA_POLY = DNA polymerase; DNA_PACK = associated with DNA packaging; AP_endo = apurinic endonuclease; RVT = reverse transcriptase; GiY-YIG_PLEs = GIY-YIG endonuclease domain of penelope-like elements; Asp_prot = aspartate proteases; SMC = Chromosome segregation ATPase; GAG = capsid protein; ENV = viral inclusion body protein; UBN = ubinuclein conserved middle domain; Dam = DNA N-6-adenine-methyltransferase; Chromo = chromo (CHRromatin Organisation MOdifier) domain.

B) Summary statistics for Pfam and CDD scans of consensus (families of) repeat sequences of *Bsal* and *Bd*, and individual occurrences of *Bsal*'s BatrLINE-1 (rnd-2 family 2), BatrLINE-2 (rnd-3 family 12) and BatrLINE-3 (rnd-4 family 12), as well as *Bsal*'s LINE, LINE/RTE-X and LTR/Gypsy non-consensus individual occurences. DNA = DNA transposon; RVT = reverse transcriptase.

C-G) Scan results for C) *Bsal*'s consensus repeat sequences, D) *Bsal*'s LINE BatrLINE-1 (rnd-2 family 2) non-consensus individual insertion sequences and E) *Bsal*'s BatrLINE-2 (rnd-3 family 12) non-consensus individual insertion sequences, F) *Bsal*'s BatrLINE-3 (rnd-4 family 12) non-consensus individual insertion sequences and G) BD's consensus repeat sequences. An e-value cutoff < 1e-3 was used for hmmsearch of Pfams and rpsblast for CDD profiles. Repeat frames refer to the 6 possible reading frames EMBOSS transeq outputs when translating the nucleotide sequence into possible protein sequences. LINEs are classified as fully functional if they have both a reverse transcriptase and one apurinic endonuclease domain. In the case of LTRs, repeat families were considered potentially mobile if they feature domains for GAG, a structural protein for virus-like particles, and for POL, which encodes an aspartic proteinase (AP), RT, and DDE integrase (INT).

Dataset S3. Repeat Induced Point mutation defective **(**RID) proteins and RNAi machinery.

A) Accession numbers and species of reference RID proteins (72) used for blastp search in *Bd* and *Bsal*, as well as for MAFFT alignment to putative C-5 cytosine methyltransferases of *Bsal*.

B) Accession numbers and species of reference RNAi proteins used for blastp search in all chytrids (based on (74) and NCBI HomoloGene search). Argo= Argonaute/piwi; RdRP = RNA dependent RNA polymerase.

C) Lowest e-value blast hits of homologs to Dicer, Argo and RdRP of all chytrids.

Dataset S4. Parameters and *p*-values of hypergeometric tests, χ^2-tests and Wilcoxon rank sum tests. For all enrichment tests, the four quadrants (Q_{SL}, Q_{LL}, Q_{LS} and Q_{SS}) are based on the 5' and 3' median $\log_{10}$ intergenic distances.

A) Number of M36 metalloprotease genes, genes coding for secreted proteins, small secreted protein (SSP) genes and core-conserved protein (CCG) genes in all 22 chytrids. SSPs are defined as either having fewer than 200 amino acids (aa; all chytrids) or proteins with fewer than 200 aa and ≥ 4 cysteines (for Rhizophydiales).

B) *p*-values of hypergeometric tests for enrichment of M36 metalloproteases, secreted proteins, core-conserved genes and small secreted proteins (defined as smaller than 200 aa and ≥4 cysteines for Rhizophydiales and only as smaller than 200 aa for the rest) in the four quadrants for all 22 chytrids. Significant *p*-values ($p_{adjusted} < 0.00063$; α-level = 0.01, $n = 16$) are highlighted in blue.

C) *p*-values for χ^2-tests for enrichment of M36 metalloproteases, secreted proteins, core-conserved genes and small secreted proteins (defined as smaller than 200 aa and ≥4 cysteines for Rhizophydiales and only as smaller than 200 aa for the rest) in the four quadrants for all 22 chytrids. Significant *p*-values ($p_{adjusted} < 0.00063$; α-level = 0.01, $n = 16$) are highlighted in blue.

D) Wilcoxon rank-sum test results for distributions of all mean upstream and downstream $\log_{10}$ intergenic regions compared to mean upstream and downstream $\log_{10}$ intergenic regions of M36 metalloproteases, secreted proteins, core-conserved genes and small secreted proteins (defined as smaller than 200 aa and ≥4 cysteines for Rhizophydiales and only as smaller than 200 aa for the rest) in all 22 chytrids. **** = 1e-04, *** = 0.001, ** = 0.01, * = 0.05, ns = 1. Significant *p*-values ($p_{adjusted} <$ 3.0303E-05; α-level = 0.01, $n = 330$) are highlighted in blue.

E) Enrichment of M36 metalloproteases, secreted proteins, core-conserved genes and small secreted proteins in *Bd* among the four quadrants was calculated by

hypergeometric tests and χ^2-tests (*p*-values shown). Significant *p*-values ($p_{adjusted} <$ 0.0005; α-level = 0.01, *n* = 20) are highlighted in blue.

F) Enrichment of BatrLINE-1, BatrLINE-2, BatrLINE-3 and BatrREP-1 and BatrREP-2 repeat families in the four quadrants in *Bsal* was calculated using hypergeometric tests and for χ^2-tests (*p*-values shown). Significant *p*-values ($p_{adjusted} < 0.0025$; α-level = 0.01, *n* = 4) are highlighted in blue.

G) Enrichment of repeat-families upstream and downstream of M36 metalloproteases in *Bsal*. *P*-values are calculated for hypergeometric tests and χ^2-tests. Repeat families were annotated according to RepeatModeller classifications. Significant *p*-values ($p_{adjusted} < 0.000083$; α-level = 0.01, *n* = 120) are highlighted in blue.

H) Enrichment of repeat-families upstream and downstream of secreted protein coding genes in *Bsal*. *P*-values are calculated for hypergeometric tests and χ^2-tests. Significant *p*-values ($p_{adjusted} < 0.00001639$; α-level = 0.01, *n* = 610) are highlighted in blue.

I) Enrichment of the 10 largest secreted tribes in the four quadrants was calculated for *Bsal*, *Bd*, *Eh* and *Hp*. *P*-values were determined using hypergeometric tests. Significant *p*-values ($p_{adjusted} < 0.00063$; α-level = 0.01, *n* = 16 (four quadrants and four species for each tribe)) are highlighted in blue.

J) Numbers of genes in each secreted Tribe and the number and names of assigned PFAMs. NA = non-applicable.

K) Details of 10 kb non-overlapping windows categorized according to internal gene quadrants. Windows with no predicted genes or only terminal genes were considered uncharacterized ($Q_{unchar.}$).

L) Enrichment and counts of upregulated genes during infection of *T. wenxineses in vivo* and *in vitro* in *Bsal* and *Bd* in the four quadrants. The *p*-values are calculated for hypergeometric tests and χ^2-tests. Significant *p*-values ($p_{adjusted} < 0.000625$; α-level = 0.01, *n* = 16) are highlighted in blue.

Dataset S5. Gene IDs for genes with a secretion signal, M36 metalloproteases, core-conserved genes (CCGs), short secreted proteins (SSPs) and upregulated, differentially expressed genes (DEGs) *in vivo* (during infection of *T. wenxineses*) and *in vitro* for *Bsal* and *Bd*. SSPs IDs are for SSPs defined as being shorter than 200 amino acids and with ≥ 4 cysteines.

Dataset S6. Quadrant enrichment and distribution on the chromosomes. The enrichment tests for the four quadrants Q_{SL}, Q_{LL}, $Q_{LS}$and Q_{SS} are based on the 5' and 3' median log_{10} intergenic distances.

A) Enrichment of genes belonging to one of the 4 quadrants on each chromosome was calculated using Hypergeometric tests, testing if there are more genes in a given quadrant on that chromosome than would be expected for the overall number of genes on the chromosome, given the number of genes in the quadrants in the entire genome. The significance level was adjusted to $p < 0.0025$ (α-level = 0.01, $n = 4$). Significant enrichments are highlighted in blue.

B) χ^2-test for goodness of fit of quadrant distribution on chromosomes. Significant deviations from the distribution of numbers of genes in quadrants from the expected 25% each are highlighted in blue. The significance level is Bonferroni adjusted to $p <$ 0000633 (α-level = 0.01, $n = 158$). Obs = observed count; Exp = expected count.

Dataset S7. Consecutive gene counts and discrete-time Markov Chain probabilities.

A) Consecutive gene counts for the four quadrants (Q_{SL}, Q_{LL}, Q_{LS} and Q_{SS}) are based on the 5' and 3' median log_{10} intergenic distances. Start and end position of the block of consecutive genes are indicated.

B) Probabilities for the number of consecutive genes found in the four quadrants. Probabilities of finding consecutive genes of length k on contigs with n genes, computed using discrete-time pattern Markov chains. Significant (Sig) *p*-values (p < 0.01) are marked with *.

C) Longest number of consecutive genes in each of the four quadrants for each chytrid.

D) Number of regions with a significant number of consecutive genes found in the four quadrants that reaches a probability of p<0.01 for all chytrids investigated. Probabilities of finding stretches of consecutive genes of length k on contigs with n genes are computed using discrete-time pattern Markov chains.

E) Significant stretches of genes belonging to Q_{LL} or Q_{SS} for each of the chytrid genomes, identified with the Markov chain approach, with and without secreted proteins. The numbers of genes in stretches of consecutive genes in the gene-sparse (Q(long-long)) and gene-rich (Q(short-short)) for each chytrid, as well as the number of stretches of consecutive genes containing secreted proteins, are listed. The difference of % of stretches of consecutive genes with secreted proteins in Q(long-long) and Q(short-short) is listed.

Dataset S8. Matching tribes of *Bsal* assemblies v2.0 and v1.0. Gene IDs of genes assigned to the respective tribes are denoted in *Bsal* v2.0 Gene ID and *Bsal* v1.0 Gene ID. Note that IDs in the same row are not homologous.

Dataset S9. M36 Metalloprotease encoding genes in the *Bsal* genome assembly v2.0 and their matching genes and classifications according to *Farrer et al.* 2017 (2). Contig numbers (terminal numbers of scaffolds), gene IDs, gene IDs in *Farrer et al.* 2017, IDs of upstream flanking genes and repeats (upstream ID), IDs of downstream flanking repeats and genes (downstream ID), classification as secreted (secreted) or not (NA), Tribes of *Bsal* assembly v2.0, the matching clades in Fig. 5 and M36 clades G1M36 or G2M36 designation (2) of all M36 metalloproteases in *Bsal* assembly v2.0.

Dataset S10. Differential expression profiles of repeat families in *Bsal*. Differential expression profiles subsets of **A)** consensus repeat families upregulated *in vivo* in *Bsal*, **B)** consensus repeat families downregulated *in vivo* in *Bsal*, **C)** individual, non-consensus insertions of LINE BatrLINE-1 upregulated *in vivo* in *Bsal* and **D)** individual, non-consensus insertions of LINE BatrLINE-1 downregulated *in vivo* in *Bsal*. logFC = $\log_2$ fold-change; logCPM = $\log_2$ counts per million; FDR = false discovery rate; TMM =Trimmed Mean of M-values.

Dataset S11. Repeatmodeller consensus sequences of repeat families flanking M36 metalloproteases upstream and downstream. Rnd-2 family 2 = BatrLINE-1; rnd-3 family 12 = BatrLINE-2; rnd-4 family 12 = BatrLINE-3; rnd-1 family 182 = BatrREP-1; rnd-1 family 405 = BatrREP-2.

Dataset S12. FASTA files of all occurrences of repeats in the genome classified as *Batr*LINE-1.

Dataset S13. FASTA files of all occurrences of repeats in the genome classified as *Batr*LINE-2.

Dataset S14. FASTA files of all occurrences of repeats in the genome classified as *Batr*LINE-3.

Dataset S15. FASTA files of all occurrences of repeats in the genome classified as *Batr*REP-1.

Dataset S16. FASTA files of all occurrences of repeats in the genome classified as *Batr*REP-2.

SI References

1. K. R. Bradnam, *et al.*, Assemblathon 2: evaluating *de novo* methods of genome assembly in three vertebrate species. *GigaScience* **2**, 10 (2013).

2. R. A. Farrer, *et al.*, Genomic innovations linked to infection strategies across emerging pathogenic chytrid fungi. *Nat. Commun.* **8** (2017).

3. T. Wicker, *et al.*, A unified classification system for eukaryotic transposable elements. *Nat. Rev. Genet.* **8**, 973–982 (2007).

4. S. van Wyk, *et al.*, The RIPper, a web-based tool for genome-wide quantification of Repeat-Induced Point (RIP) mutations. *PeerJ* **7**, e7447 (2019).

5. Z. A. Lewis, *et al.*, Relics of repeat-induced point mutation direct heterochromatin formation in *Neurospora crassa*. *Genome Res.* **19**, 427–437 (2009).

6. J. K. Hane, R. P. Oliver, RIPCAL: a tool for alignment-based analysis of repeat-induced point mutations in fungal genomic sequences. *BMC Bioinformatics* **9**, 478 (2008).

7. B. S. Margolin, *et al.*, A methylated *Neurospora* 5S rRNA pseudogene contains a transposable element inactivated by repeat-induced point mutation. *Genetics* **149**, 1787–1797 (1998).

8. A. M. Lambowitz, S. Zimmerly, Group II Introns: Mobile Ribozymes that Invade DNA. *Cold Spring Harb. Perspect. Biol.* **3**, a003616–a003616 (2011).

9. B. Piégu, S. Bire, P. Arensburger, Y. Bigot, A survey of transposable element classification systems – A call for a fundamental update to meet the challenge of their diversity and complexity. *Mol. Phylogenet. Evol.* **86**, 90–109 (2015).

10. B. Schwessinger, J. P. Rathjen, "Extraction of High Molecular Weight DNA from Fungal Rust Spores for Long Read Sequencing" in *Wheat Rust Diseases. Methods in Molecular Biology*, vol 1659, S. Periyannan, Ed. (Humana Press, 2017), pp. 49–57.

11. B. Schwessinger, High quality DNA from Fungi for long read sequencing e.g. PacBio. *protocols.io* (2019) https:/doi.org/dx.doi.org/10.17504/protocols.io.2yfgftn.

12. R. R. Wick, L. M. Judd, C. L. Gorrie, K. E. Holt, Completing bacterial genome assemblies with multiplex MinION sequencing. *Microb. Genomics* **3**, 1–7 (2017).

13. W. De Coster, S. D'Hert, D. T. Schultz, M. Cruts, C. Van Broeckhoven, NanoPack: Visualizing and processing long-read sequencing data. *Bioinformatics* **34**, 2666–2669 (2018).

14. S. Koren, *et al.*, Canu:scalable and accurate long-read assembly via adaptive k-mer weighting and repeat separation. *Genome Res.* **27**, 722–736 (2017).

15. R. Vaser, M. Šikić, Yet another de novo genome assembler. *bioRxiv* (2019) https:/doi.org/10.1101/656306 (November 21, 2022).

16. B. J. Walker, *et al.*, Pilon: An integrated tool for comprehensive microbial variant detection and genome assembly improvement. *PLoS ONE* **9** (2014).

17. A. Mikheenko, A. Prjibelski, V. Saveliev, D. Antipov, A. Gurevich, Versatile genome assembly evaluation with QUAST-LG. *Bioinformatics* **34**, i142–i150 (2018).

18. G. Parra, K. Bradnam, I. Korf, CEGMA: A pipeline to accurately annotate core genes in eukaryotic genomes. *Bioinformatics* **23**, 1061–1067 (2007).

19. M. Seppey, M. Manni, E. M. Zdobnov, "BUSCO: Assessing Genome Assembly and Annotation Completeness" in *Gene Prediction: Methods and Protocols*, M. Kollmar, Ed. (Springer Science+Business Media, 2019), pp. 227–245.

20. M. Hunt, *et al.*, REAPR: A universal tool for genome assembly evaluation. *Genome Biol.* **14** (2013).

21. S. Kurtz, *et al.*, Versatile and open software for comparing large genomes. *Genome Biol.* **5** (2004).

22. T. R. Ranallo-Benavidez, K. S. Jaron, M. C. Schatz, GenomeScope 2.0 and Smudgeplot for reference-free profiling of polyploid genomes. *Nat. Commun.* **11**, 1432 (2020).

23. M. Kokot, M. Długosz, S. Deorowicz, KMC 3: counting and manipulating k-mer statistics. *Bioinformatics* **33**, 2759–2761 (2017).

24. K. J. Hoff, A. Lomsadze, M. Borodovsky, M. Stanke, "Whole-genome annotation with BRAKER" in *Methods in Molecular Biology*, 1962nd Ed., M. Kollmar, Ed. (Humana, New York, NY., 2019), pp. 62–95.

25. H. Li, *et al.*, The Sequence Alignment/Map format and SAMtools. *Bioinformatics* **25**, 2078–2079 (2009).

26. D. W. Barnett, E. K. Garrison, A. R. Quinlan, M. P. Střmberg, G. T. Marth, Bamtools: A C++ API and toolkit for analyzing and managing BAM files. *Bioinformatics* **27**, 1691–1692 (2011).

27. B. Buchfink, C. Xie, D. H. Huson, Fast and sensitive protein alignment using DIAMOND. *Nat. Methods* **12**, 59–60 (2014).

28. A. Lomsadze, P. D. Burns, M. Borodovsky, Integration of mapped RNA-Seq reads into automatic training of eukaryotic gene finding algorithm. *Nucleic Acids Res.* **42**, 1–8 (2014).

29. M. Stanke, M. Diekhans, R. Baertsch, D. Haussler, Using native and syntenically mapped cDNA alignments to improve de novo gene finding. *Bioinformatics* **24**, 637–644 (2008).

30. A. Bairoch, R. Apweiler, The SWISS-PROT protein sequence database and its supplement TrEMBL in 2000. *Nucleic Acids Res.* **28**, 45–48 (2000).

31. M. Kanehisa, S. Goto, KEGG: kyoto encyclopedia of genes and genomes. *Nucleic Acids Res.* **28**, 27–30 (2000).

32. R. D. Finn, J. Clements, S. R. Eddy, HMMER web server: interactive sequence similarity searching. *Nucleic Acids Res.* **39**, W29–W37 (2011).

33. R. D. Finn, *et al.*, Pfam: the protein families database. *Nucleic Acids Res.* **42**, D222–D230 (2014).

34. T. M. Lowe, S. R. Eddy, tRNAscan-SE: a program for improved detection of transfer RNA genes in genomic sequence. *Nucleic Acids Res.* **25**, 955–964 (1997).

35. K. Lagesen, *et al.*, RNAmmer: consistent and rapid annotation of ribosomal RNA genes. *Nucleic Acids Res.* **35**, 3100–3108 (2007).

36. D. H. Haft, J. D. Selengut, O. White, The TIGRFAMs database of protein families. *Nucleic Acids Res.* **31**, 371–373 (2003).

37. R. D. Finn, *et al.*, Pfam: the protein families database. *Nucleic Acids Res.* **42**, D222–D230 (2014).

38. T. N. Petersen, S. Brunak, G. von Heijne, H. Nielsen, SignalP 4.0: discriminating signal peptides from transmembrane regions. *Nat. Methods* **8**, 785–786 (2011).

39. A. Krogh, B. Larsson, G. von Heijne, E. L. Sonnhammer, Predicting transmembrane protein topology with a Hidden Markov Model: application to complete genomes. *J. Mol. Biol.* **305**, 567–580 (2001).

40. R. C. Edgar, MUSCLE: a multiple sequence alignment method with reduced time and space complexity. *BMC Bioinformatics* **5**, 113 (2004).

41. S. Capella-Gutiérrez, J. M. Silla-Martínez, T. Gabaldón, trimAl: a tool for automated alignment trimming in large-scale phylogenetic analyses. *Bioinforma. Oxf. Engl.* **25**, 1972–1973 (2009).

42. A. Stamatakis, RAxML-VI-HPC: maximum likelihood-based phylogenetic analyses with thousands of taxa and mixed models. *Bioinformatics* **22**, 2688–2690 (2006).

43. I. Letunic, P. Bork, Interactive Tree Of Life (iTOL) v5: an online tool for phylogenetic tree display and annotation. *Nucleic Acids Res.* **49**, W293–W296 (2021).

44. R. Farrer, Homolaphlyctis polyrhiza annotation GFF3. figshare. Dataset. (2016).

45. I. V. Grigoriev, *et al.*, MycoCosm portal: gearing up for 1000 fungal genomes. *Nucleic Acids Res.* **42**, D699–D704 (2014).

46. S. R. Ahrendt, *et al.*, Leveraging single-cell genomics to expand the fungal tree of life. *Nat. Microbiol.* **3**, 1417–1428 (2018).

47. Y. Chang, *et al.*, Phylogenomic Analyses Indicate that Early Fungi Evolved Digesting Cell Walls of Algal Ancestors of Land Plants. *Genome Biol. Evol.* **7**, 1590–1601 (2015).

48. S. J. Mondo, *et al.*, Widespread adenine N6-methylation of active genes in fungi. *Nat. Genet.* **49**, 964–968 (2017).

49. S. E. Mozley-Standridge, P. M. Letcher, J. E. Longcore, D. Porter, D. R. Simmons, Cladochytriales--a new order in Chytridiomycota. *Mycol. Res.* **113**, 498–507 (2009).

50. R. A. Farrer, Synima: a synteny imaging tool for annotated genome assemblies. *BMC Bioinformatics* **18**, 507 (2017).

51. L.-T. Nguyen, H. A. Schmidt, A. von Haeseler, B. Q. Minh, IQ-TREE: A Fast and Effective Stochastic Algorithm for Estimating Maximum-Likelihood Phylogenies. *Mol. Biol. Evol.* **32**, 268–274 (2015).

52. D. Darriba, G. L. Taboada, R. Doallo, D. Posada, ProtTest 3: fast selection of best-fit models of protein evolution. *Bioinformatics* **27**, 1164–1165 (2011).

53. J. M. Flynn, *et al.*, RepeatModeler2 for automated genomic discovery of transposable element families. *Proc. Natl. Acad. Sci. U. S. A.* **117**, 9451–9457 (2020).

54. G. Benson, Tandem repeats finder: A program to analyze DNA sequences. *Nucleic Acids Res.* **27**, 573–580 (1999).

55. A. L. Price, N. C. Jones, P. A. Pevzner, De novo identification of repeat families in large genomes. *Bioinformatics* **21**, 351–358 (2005).

56. A. Smit, R. Hubley, P. Green, RepeatMasker Open-4.0. (2015).

57. J. T. Robinson, *et al.*, Integrative genomics viewer. *Nat. Biotechnol.* **29**, 24–26 (2011).

58. G. N. Wilkinson, C. E. Rogers, Symbolic Description of Factorial Models for Analysis of Variance. *J. R. Stat. Soc. Ser. C Appl. Stat.* **22**, 392–399 (1973).

59. P. Rice, I. Longden, A. Bleasby, EMBOSS: the European Molecular Biology Open Software Suite. *Trends Genet. TIG* **16**, 276–277 (2000).

60. J. Mistry, *et al.*, Pfam: The protein families database in 2021. *Nucleic Acids Res.* **49**, D412–D419 (2021).

61. S. R. Eddy, Accelerated Profile HMM Searches. *PLOS Comput. Biol.* **7**, e1002195 (2011).

62. S. Lu, *et al.*, CDD/SPARCLE: the conserved domain database in 2020. *Nucleic Acids Res.* **48**, D265–D268 (2020).

63. C. Camacho, *et al.*, BLAST+: architecture and applications. *BMC Bioinformatics* **10**, 421 (2009).

64. A. Muszewska, K. Steczkiewicz, M. Stepniewska-Dziubinska, K. Ginalski, Cut-and-Paste Transposons in Fungi with Diverse Lifestyles. *Genome Biol. Evol.* **9**, 3463–3477 (2017).

65. A. Muszewska, K. Steczkiewicz, M. Stepniewska-Dziubinska, K. Ginalski, Transposable elements contribute to fungal genes and impact fungal lifestyle. *Sci. Rep.* **9**, 4307 (2019).

66. H.-G. Drost, LTRpred: *de novo* annotation of intact retrotransposons. *J. Open Source Softw.* **5**, 2170 (2020).

67. M. Naish, *et al.*, The genetic and epigenetic landscape of the *Arabidopsis* centromeres. *Science* **374**, eabi7489 (2021).

68. K. Katoh, D. M. Standley, MAFFT Multiple Sequence Alignment Software Version 7: Improvements in Performance and Usability. *Mol. Biol. Evol.* **30**, 772–780 (2013).

69. S. Kalyaanamoorthy, B. Q. Minh, T. K. F. Wong, A. von Haeseler, L. S. Jermiin, ModelFinder: fast model selection for accurate phylogenetic estimates. *Nat. Methods* **14**, 587–589 (2017).

70. D. T. Hoang, O. Chernomor, A. von Haeseler, B. Q. Minh, L. S. Vinh, UFBoot2: Improving the Ultrafast Bootstrap Approximation. *Mol. Biol. Evol.* **35**, 518–522 (2018).

71. M. Blum, *et al.*, The InterPro protein families and domains database: 20 years on. *Nucleic Acids Res.* **49**, D344–D354 (2021).

72. B. van de Vossenberg, *et al.*, Comparative genomics of chytrid fungi reveal insights into the obligate biotrophic and pathogenic lifestyle of *Synchytrium endobioticum*. *Sci. Rep.* **9**, 8672 (2019).

73. A. M. Waterhouse, J. B. Procter, D. M. A. Martin, M. Clamp, G. J. Barton, Jalview Version 2—a multiple sequence alignment editor and analysis workbench. *Bioinformatics* **25**, 1189–1191 (2009).

74. S.-J. Lee, M. Kong, P. Harrison, M. Hijri, Conserved Proteins of the RNA Interference System in the Arbuscular Mycorrhizal Fungus *Rhizoglomus irregulare* Provide New Insight into the Evolutionary History of Glomeromycota. *Genome Biol. Evol.* **10**, 328–343 (2018).

75. R. A. Farrer, *et al.*, Multiple emergences of genetically diverse amphibian-infecting chytrids include a globalized hypervirulent recombinant lineage. *Proc. Natl. Acad. Sci. U. S. A.* **108**, 18732–18736 (2011).

76. S. J. O'Hanlon, *et al.*, Recent Asian origin of chytrid fungi causing global amphibian declines. *Science* **360**, 621–627 (2018).

77. R. A. Farrer, *et al.*, Chromosomal copy number variation, selection and uneven rates of recombination reveal cryptic genome diversity linked to pathogenicity. *PLOS Genet* **9**, e1003703 (2013).

78. A. McKenna, *et al.*, The Genome Analysis Toolkit: a MapReduce framework for analyzing next-generation DNA sequencing data. *Genome Res.* **20**, 1297–1303 (2010).

79. K. Voss, G. V. der Auwera, J. Gentry, Full-stack genomics pipelining with GATK4 + WDL + Cromwell in *18th Annual Bioinformatics Open Source Conference (BOSC 2017)*, (2017).

80. H. Li, Aligning sequence reads, clone sequences and assembly contigs with BWA-MEM. *ArXiv13033997 Q-Bio* (2013) (May 29, 2018).

81. Z. Yang, PAML 4: phylogenetic analysis by maximum likelihood. *Mol. Biol. Evol.* **24**, 1586–1591 (2007).

82. Z. Yang, R. Nielsen, Estimating synonymous and nonsynonymous substitution rates under realistic evolutionary models. *Mol. Biol. Evol.* **17**, 32–43 (2000).

83. B. J. Haas, *et al.*, *De novo* transcript sequence reconstruction from RNA-Seq: reference generation and analysis with Trinity. *Nat. Protoc.* **8** (2013).

84. B. Langmead, S. L. Salzberg, Fast gapped-read alignment with Bowtie 2. *Nat. Methods* **9**, 357–359 (2012).

85. B. Li, C. N. Dewey, RSEM: accurate transcript quantification from RNA-Seq data with or without a reference genome. *BMC Bioinformatics* **12**, 323 (2011).

86. M. D. Robinson, D. J. McCarthy, G. K. Smyth, EdgeR: a Bioconductor package for differential expression analysis of digital gene expression data. *Bioinformatics* **26**, 139–140 (2010).

87. T. R. Sewell, J. Longcore, M. C. Fisher, *Batrachochytrium dendrobatidis*. *Trends Parasitol.* **37**, 933–934 (2021).
